# Genetic Monitoring of Brown Trout Released Into a Novel Environment: Establishment and Genetic Impact on Natural Populations

**DOI:** 10.1111/eva.70084

**Published:** 2025-02-27

**Authors:** Anastasia Andersson, Sara Kurland, Sten Karlsson, Nils Ryman, Linda Laikre

**Affiliations:** ^1^ Department of Zoology Stockholm University Stockholm Sweden; ^2^ Department of Earth Sciences Natural Resources and Sustainable Development (NRHU), Uppsala University Visby Sweden; ^3^ Norwegian Institute for Nature Research (NINA) Trondheim Norway

**Keywords:** indicators for genetic diversity, monitoring genetic diversity, population genetics, *Salmo trutta*

## Abstract

Translocations are carried out either unintentionally or intentionally for conservation or management reasons. In both cases, translocated populations may genetically impact natural populations via introgression. Understanding how genetic background may affect an establishment in a novel environment and the potential risks for native populations is important for biodiversity conservation. Here, using a panel of 96 SNPs, we monitor the establishment of two genetically and ecologically distinct brown trout populations released into a mountain lake system in central Sweden where trout did not occur prior to the release. The release was carried out in 1979, and we monitor the establishment over the first three decades (5–6 generations) in seven lakes downstream of the release site. We find that extensive hybridization has occurred, and genes from both populations exist in all lakes examined. Genes from the population that was nonmigratory in its native environment have remained to a higher degree in the area close to the release site, while genes from the population that was more migratory in its native habitat have spread further downstream. All established populations exhibit higher levels of genetic diversity than the released populations. Natural, stream‐resident brown trout populations occur ~15 km downstream of the release site and below a waterfall that acts as an upstream migration barrier. Released fish have spread genes to these populations but with low introgression rates of 3%–8%. Recently adopted indicators for monitoring genetic diversity were partly able to detect this introgression, emphasizing the usefulness of genetic indicators in management. The SNP panel used in this study provides a similar picture as previously used allozymes, showing that older marker systems with fewer loci may still be useful for describing the population structure.

## 1 Introduction

Understanding the biological dynamics following populations establishing in areas where they have not occurred previously is of growing importance because several processes increase such establishments (Gordeeva and Salmenkova 2011). Climate change, habitat loss, and ecosystem degradation drive populations out of their native distribution ranges, and new territories are sometimes colonized (Dupoué et al. 2020; Carvalho et al. 2019; Hampe et al. 2013). Similarly, the deliberate anthropogenic release of animals or plants into areas where they have not occurred previously is carried out for many species, e.g., to increase harvest or for conservation purposes (Laikre et al. 2006, 2010). Translocations are expected to be increasingly common following climate change and as human land use practices continue to alter habitats (Muhlfeld et al. 2009, 2019; Pearman et al. 2024). The movement of populations poses genetic threats to species worldwide (Ceballos et al. 2017; Allendorf et al. 2001).

Several key questions regarding the consequences of relocating populations into new areas remain. One such question relates to the effect of the genetic background of the relocated populations for establishment in a novel environment (Hampe et al. 2013; Willoughby et al. 2018). Another question concerns the frequency and effect of hybridization between non‐native and native populations (Allendorf et al. 2001; Vlahiotis et al. 2023), which can have detrimental effects on the native ones. One example of this is the widespread genetic introgression of escaped farmed Atlantic salmon ( *Salmo salar* ) into wild populations, resulting in fitness loss in the wild (Karlsson et al. 2016; Palm et al. 2021). Understanding these aspects should advance our knowledge on the spread of translocated populations and their potential impact on native populations. Such knowledge is also crucial for management and conservation planning to mitigate unintended impacts resulting from translocations (Vlahiotis et al. 2023). Thus, empirical evaluation of the genetic effects of human‐mediated releases in the wild is essential.

The brown trout ( *Salmo trutta* ) is one of the most frequently translocated and stocked species (Filipsson 1994; Tammi et al. 2003; Hesthagen and Sandlund 2004; Laikre et al. 2006). It is endemic throughout Europe (Jonsson and Jonsson 2011) and carries an important ecological role, particularly in the mountains of Scandinavia, where it is a top predator and often the only or one of just a few fish species (Frank et al. 2011; Marco‐Rius et al. 2013). In these ecosystems, it is common to find substantial genetic differentiation between neighboring lakes, and such between‐population differences account for the majority of the biological diversity within the species (Ryman 1983). Thus, it is imperative to safeguard this kind of intraspecific biodiversity as it constitutes the basis for adaptation and long‐term survival of the brown trout in the subarctic.

Here, we focus on a case of an intentional human‐mediated release of two brown trout populations (A and B; see Section 2.2) into the wild in a common garden experiment and monitor their establishment over three decades using a panel of 96 SNPs. The fish were released into a lake system previously void of brown trout. The progeny spread downstream and came into contact with native populations after passing a waterfall preventing upstream migration. We study microevolutionary processes following the release, linking the observations to conservation management and policy implementation. Our main objectives were to (i) examine the pattern of hybridization between populations A and B over multiple generations in the lakes originally void of brown trout, (ii) monitor the potential introgression of genes from the released populations into native, stream‐resident brown trout occurring below the waterfall that prevents upstream migration, and (iii) apply new indicators for genetic diversity monitoring to examine the extent to which they could detect an impact of the release on the native populations. Allozyme data at about 15 loci were also available for the fish scored at 96 SNPs, and an additional objective was (iv) to examine the extent to which these two marker systems provide similar results.

## 2 Materials and Methods

### 2.1 Study Area

**FIGURE 1:**
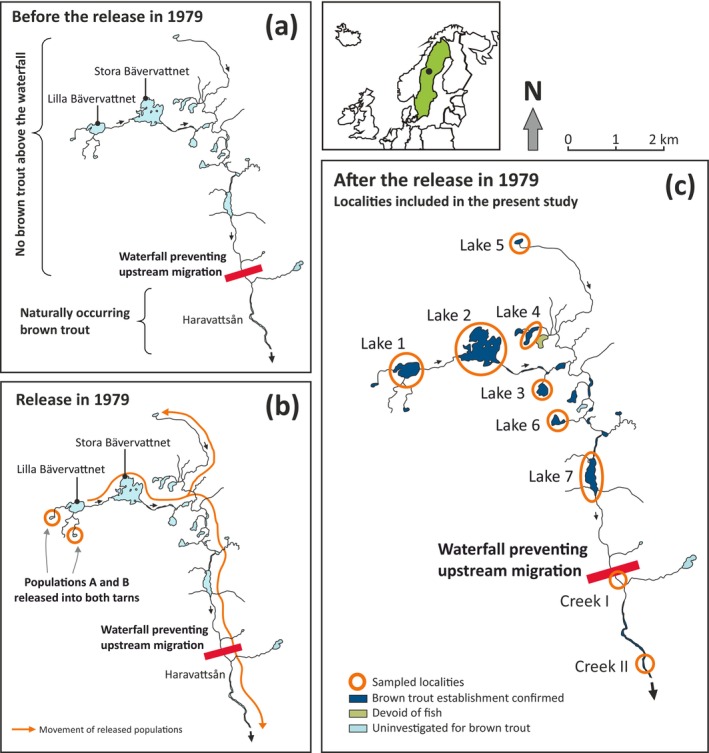
Design of the current study. Panel (a) shows the water system before the release of populations A and B. Panel (b) illustrates the release and subsequent movement of released populations throughout the water system as indicated by the orange arrows, and panel (c) depicts the sampling efforts for the current study. The scale in the top right corner refers to panel (c).

The study area is part of the Hotagen Nature Reserve, Jämtland County, central Sweden (Figure 1), located in the uppermost part of the drainage basin of River Indalsälven, a major river flowing into the Baltic Sea. It belongs to the subalpine, mountainous area of western Sweden, located just below the tree line at an elevation of 600–700 m (see Table S1 for geographic coordinates, local names, and their designations in the present paper).

The characteristics of the local topography create a situation such that the waters above the waterfall in Creek Haravattsån (Figure 1) represent a naturally “closed region” that prevents fish from immigrating from the outside into the sub‐drainage located above this waterfall. First, there is a drainage divide just north and west of the area shown in Figure 1, and there are no other lakes flowing into the area above the waterfall. Further, the waterfall itself prevents upstream migration, and fish cannot enter the upstream waters without human support.

The area above the waterfall was originally void of brown trout. Arctic char ( *Salvelinus alpinus* ) was the only fish present, but natural populations of brown trout occur below the waterfall (Figure 1a). In 1979, fry from two populations of brown trout (A and B) were introduced in the uppermost part above the waterfall in a common garden experiment. This introduction was originally aimed at examining phenotypic differences between A and B in a common environment. Such comparisons were possible in the first two generations, the P generation and the first offspring generation (F1), because A and B were homozygous for different alleles at an allozyme locus (*G3PDH‐2*; Palm and Ryman 1999).

The progeny of the released fish spread and established self‐sustaining populations in the creeks and lakes downstream from the release site. Some fish also migrated over the waterfall into areas occupied by naturally occurring brown trout (Figure 1b). We have continued to periodically sample trout above and below the waterfall and used the material for addressing questions relating to the spread of the two populations. To some extent, the present study uses samples that have also been included in previous publications (Table 1).

**TABLE 1:** Sampling localities and SNP analysis sample sizes of this study.

Locality/Population	Sample size (*n*)	Sampling year	Included in previous publications
Introduced population A (baseline)	110	1988–1999	Palm and Ryman 1999 (*n* = 84; allozymes) Kurland et al. 2022 (*n* = 50; WGS pool‐seq)
Introduced population B (baseline)	108	1988–1992	Palm and Ryman 1999 (*n* = 93; allozymes) Kurland et al. 2022 (*n* = 50; WGS pool‐seq)
**Total introduced populations**	218		
Lake 1	60	2011	Kurland et al. 2022 (*n* = 50; WGS pool‐seq)
Lake 2	59	2011	
Lake 3	57	2007	
Lake 4	57	2007	
Lake 5	60	2015	
Lake 6	26	2007	
Lake 7	59	2011	Kurland et al. 2022 (*n* = 50; WGS pool‐seq)
**Total established**	378		
Creek I pre‐release (baseline)	135	1980–1988	Palm et al. 2003 (*n* = 135; allozymes)
Creek I 1999	60	1999	
Creek I 2011	60	2011	
**Total Creek I**	255		
Creek II pre‐release (baseline)	127	1984–1991	Palm et al. 2003 (*n* = 127; allozymes)
Creek II 1999	60	1999	
Creek II 2011	60	2011	
**Total Creek II**	247		
**TOTAL**	1098		

*Note:* Some of the fish have also been included in previous publications from this water system using different types of markers (allozymes, whole genome sequencing (WGS)). Reference, overlapping sample sizes, and types of genetic markers used are indicated in the rightmost column.

The introduction of brown trout has resulted in a reduction in the Arctic char population in the lakes above the waterfall. From about the year 2000 and onward, the vast majority of fish caught by gillnetting are brown trout, but the char has not disappeared. Fishing was banned after the release event, and no releases occur in the water system. Our present study is distinct from previous publications on this system in addressing questions relating to hybridization between A and B above the waterfall beyond the F1 generation and to introgression from A and B into the natural populations below the waterfall.

### 2.2 Released Brown Trout Populations

In 1979, a total of 500 fry from each of A and B were released into each of two tarns in the western part of the study area (i.e., a total release of *n* = 1000 fry from each population; Figure 1b). The released fry were produced from parents from the two populations selected to ensure contrasting homozygosity at one allozyme locus (*G3PDH‐2*) that was used as a marker to identify the populations during the initial phase of the establishment process. Populations A and B were fixed for the *100* and the *50* alleles, respectively, and showed large allele frequency differences at several allozyme loci with an overall *F*_ST_ ≈ 0.40 (Palm et al. 2003; Wennerström 2010). Approximately 100 spawners were used to produce population A fry and 17 to produce population B fry. A detailed description is provided by Palm and Ryman (1999).

In addition to the genetic differences, populations A and B also exhibited divergent morphological and ecological characteristics in their native habitats. Population A originated from Lake Kallsjön, a large lake (c. 160 km^2^) located c. 100 km west of the study area. These fish were large‐sized (≥ 1 kg), slow‐growing, and piscivorous. They matured at a relatively old age and had a tendency for long‐distance migration (Ryman et al. 1986). Population B originated from two small (< 1 km^2^), closely connected lakes, Lakes Fälpfjälltjärnarna, located about 10 km north of the study area. These lakes have similar ecological characteristics as those in the release area. The trout in Lakes Fälpfjälltjärnarna were small (0.2–0.3 kg), early maturing, and predominantly insectivorous (Ryman et al. 1986). The lakes from which populations A and B were obtained are separated by more than 500 km waterway; thus, it is likely that the populations have been isolated since the last glaciation period (c. 5000–9000 years ago; Palm and Ryman 1999).

### 2.3 Sampling

Following the release in 1979, the lakes above the waterfall were inhabited only by populations A and B, and the creek below the waterfall harbored natural populations as well as immigrants from A and B (in addition to Arctic char; see Section 2.1). Repeated sampling with varying intensities (gillnetting and/or angling) has been conducted above and below the waterfall since the early 1980s (Figure 1c). The two collection sites in Creek Haravattsån were sampled from 1980. In contrast, sampling in the lakes above the waterfall was not initiated until 1988 when abundant reproduction of the released fish had been confirmed. Frozen tissue samples from all sampled individuals have been stored in a tissue bank at the Department of Zoology, Stockholm University, maintained by N.R. and L.L. We used samples from this tissue bank for the present study. Following the same procedures throughout the project, individual data on body length, sex, age (from otolith readings), and maturity stage based on gonadal development indicating whether or not the individual would have spawned in the year of sampling (the sampling occurred 1–2 months prior to the spawning period) were also recorded.

Questions regarding establishment and hybridization between populations A and B were addressed using samples from the seven lakes above the waterfall. Samples from below the waterfall were used to examine issues relating to introgression from A and B into the natural populations. Sample sizes and sampling years are provided in Table 1 along with abbreviated designations of the sampled localities (see also Figure 1c).

### 2.4 Genotyping

The project was initiated before the genomic era and relied initially on electrophoretically detectable allozyme loci as genetic markers. Most of the samples collected over the years (early 1980s through present) have routinely been genotyped at about 15 allozyme loci (for details see e.g. Palm et al. 2003; Jorde and Ryman 1996; Wennerström 2010). All samples that have been scored for the 96 SNPs in this study also had allozyme genotypes available, and in some situations, we checked the conformity between the pictures provided by these two sets of markers.

DNA extraction from muscle tissue and genotyping at the 96 SNP loci was carried out as described by Andersson et al. (2022). The 96 SNPs are distributed across the whole genome, with 1–3 SNPs (separated by a minimum of > 190,000 bases) located on each of the 40 chromosomes (Saha et al. 2022, their Figure S1).

### 2.5 Identifying Fish for SNP Baselines

Assessing hybridization patterns between A and B required allele frequencies for fish representing the two populations. Samples from the released fry were not available, and relevant “baseline” data had to be obtained from fish collected during the initial phase of the sampling efforts. To identify fish that could be considered representing “pure” A and B from the first two generations (P and F1), we used information on sampling site and year, age, and allozyme genotype (contrasting homozygosity at the *G3PDH‐2* locus). In this way, we could classify fish as being either released (P generation) or produced from within‐population matings in the P generation, resulting in homozygous progeny belonging to the F1 generation. We selected a total of 110 and 108 individuals from the A and B populations, respectively (Table 1).

For estimating introgression rates from A and B into the creek populations, we needed allele frequency baseline data from the creek localities before the release. As baselines for Creek I, just below the waterfall (*n* = 135; Table 1), and Creek II, further downstream (*n* = 127), we selected fish from each locality using similar criteria as Palm et al. (2003). That is, we (i) eliminated cohorts born after 1984 and (ii) removed from the remaining data set fish identified as potential immigrants from A or B or their first‐generation hybrids on the basis of their allozyme genotype.

To check the classification of fish selected as baseline individuals above and below the waterfall, we examined them in structure (v.2.3.4; Pritchard et al. 2000; Falush et al. 2003) analyses based exclusively on the 96 SNPs. We saw no indications of misclassifications, and a detailed description of these analyses is provided in Appendix S1.

### 2.6 Assessing Hybridization Rates Above the Waterfall

Hybridization rate between populations A and B above the waterfall was estimated as their genetic contribution to the populations residing in the seven lakes. We used structure to assign each of the 378 descendants (Table 1) to populations A and B using the baseline data (*n* = 110 and *n* = 108 for A and B, respectively; see Section 2.5) and applying the structure settings detailed in Appendix S1. The analysis yielded an assignment probability (*Q*) for each individual, i.e., the probability of belonging to a certain cluster (A or B). The *Q* values can also be interpreted as the proportion of genes originating from each cluster.

To validate our structure results, we also conducted a dapc analysis (Jombart et al. 2010), implemented in the adegenet package (v 2.1.5; Jombart 2008; Jombart and Ahmed 2011) in R (v.4.1.2; R Core Team 2021). The input data were the same as in the structure analysis, and the optimal number of genetic clusters (*K*) was assessed using the *find.clusters* function (a detailed description of this analysis is provided in Appendix S2).

### 2.7 Estimating Genetic Diversity, Divergence, and Effective Population Size

We quantified genetic diversity expressed as expected and observed heterozygosity (*H*_E_ and *H*_O_), allelic richness (*A*_R_), and the average number of alleles per locus (*A*_n_) using genalex (v.6.5 Peakall and Smouse 2006, 2012; *H*_E_, *H*_O_, *A*_n_) and fstat (v.2.9.4; Goudet 2003; *A*_R_ ).

Spatial and temporal genetic differentiation (*F*_ST_; Weir and Cockerham 1984) was estimated and tested using genepop (v.4.3; Raymond and Rousset 1995; Rousset 2008), as were deviations from Hardy–Weinberg proportions within lakes. The relationship among samples was illustrated with a neighbor‐joining phylogenetic tree based on Nei's *D*_a_ distance, constructed in poptree2 (Takezaki et al. 2010).

We estimated the effective population size (*N*_e_) with the linkage disequilibrium method (*N*_eLD_; Waples and Do 2010) using neestimator v.2.1 (Do et al. 2014), applying the model that assumes random mating. In the creek localities, we also estimated *N*_e_ with the temporal method implemented in tempofs (*N*_eV_; sampling plan II; Jorde and Ryman 1996, 2007) because temporally separated samples were available for these localities.

### 2.8 Genotypic–Phenotypic Associations in Established Populations

We examined potential associations between the phenotypic traits of migratory behavior, body size, and spawning frequency and genetic contribution from the released populations. The association between the migration distance and the genetic background of fish from the seven lakes (*n* = 378; Table 1) was tested with a linear regression analysis. We used the assignment probability to population A (*Q*_A_) from structure as the response variable and the approximate distance between the release site and the site of collection as the predictor.

For body size, we used ANCOVAs (statistica v.7.1; StatSoft Inc 2005) to control for other factors potentially affecting this trait (e.g., age, sex, and lake). We divided the fish into three genetic groups based on their assignment probability (*Q*_A_) to A. Individuals with *Q*_A_ ≥ 0.75 were classified as “A”, *Q*_A_ = 0.74–0.26 resulted in the classification as “AB”, and those with *Q*_A_ ≤ 0.25 were classified as “B”. Genetic group, lake, and sex were used as explanatory factors, and age was used as a covariate. We conducted the analyses both including the established populations only (*n* = 378; Table 1) and using a combination of the established populations and the baseline fish (*n* = 378 + 218 = 596; Table 1).

Finally, the spawning frequency in different genetic groups in the seven lakes (*n* = 378; Table 1) was compared using a Chi‐square contingency test.

### 2.9 Examining the Genetic Impact on Native Downstream Populations

We estimated the rates of migration over the waterfall into the natural populations in Creek Haravattsån (Figure 1) and rates of introgression via hybridization. This was done by letting structure assign each fish collected in Creek I and Creek II in 1999 and 2011 (total *n* = 4 × 60 = 240; Table 1) to the four groups representing populations A and B, pre‐release Creek I, and pre‐release Creek II based on baseline data for these groups (details provided in Appendix S1). The resulting *Q* values were used to estimate genetic introgression as the average proportion of genes originating from populations A and B (*Q*_A_ and *Q*_B_).

### 2.10 Applying Indicators for Genetic Diversity to Evaluate the Impact of Released Trout

We tested whether newly adopted indicators to monitor genetic diversity in Sweden (described in Andersson et al. 2022) would detect the genetic impact of the release on the native creek populations. In brief, three indicators are used: Indicator ∆*H* measures within‐population diversity, indicator *N*_e_ assesses effective population size, and indicator ∆*F*_ST_ quantifies change in genetic differentiation among populations over time. The ∆*H* indicator includes expected and observed heterozygosity (*H*_E_ and *H*_O_), allelic richness (*A*_R_), and the average number of alleles per locus (*A*_n_).

With respect to the amount of genetic change, a “traffic light” system is applied to the indicator values where green = acceptable, yellow = warning, and red = alarm. Observed genetic shifts over the study period (here about 30 years) are converted into the expected shift over 100 years assuming a constant rate of change. Threshold values for ∆*H* are related to the rate of diversity loss, so that retention of ≥ 95% of diversity over 100 years = green/acceptable, and < 75% of diversity retention over 100 years = red/alarm (cf. Allendorf and Ryman 2002). Retention of 75%–94% of diversity over 100 years, or a significant diversity increase coupled with information on anthropogenic activities, results in yellow/warning.

The threshold values for *N*_e_ follow the “50/500 rule” of Franklin (1980), suggesting *N*_e_ > 50 for short‐term conservation and *N*_e_ > 500 for long‐term. Thus, *N*_e_ > 500 = green/acceptable, 50 < *N*_e_ < 500 = yellow/warning, and *N*_e_ < 50 = red/alarm. Threshold values for ∆*F*_ST_ are as follows: No or minor change in *F*_ST_ between populations = green/acceptable, ∆*F*_ST_ reflecting a 25%–50% reduction or 50%–100% increase in genetic exchange = yellow/warning, and ∆*F*_ST_ reflecting a > 50% reduction or > 100% increase in genetic exchange = red/alarm (Andersson et al. 2022). We tested for genetic diversity changes over time within and between populations on a locus‐by‐locus basis using the nonparametric Wilcoxon matched pairs test as well as Student's *t* test for paired samples in statistica (see Nei 1987, 184).

## 3 Results

Our results indicate that extensive hybridization between the released populations has taken place. Populations A and B showed substantial genetic differentiation (*F*_ST_ > 0.4 for our present SNPs (Appendix S1) as well as for allozymes (Wennerström 2010)) when they were released into the water system. This has now changed into a situation where *F*_ST_ among the seven lakes is only about 0.03 and where almost all individuals representing the contemporary samples appear to carry genes from both populations A and B. Genes from the released populations have also spread to the native ones below the waterfall, although the level of introgression appears to be limited.

### 3.1 Establishment of, and Hybridization Between, Released Populations

**FIGURE 2:**
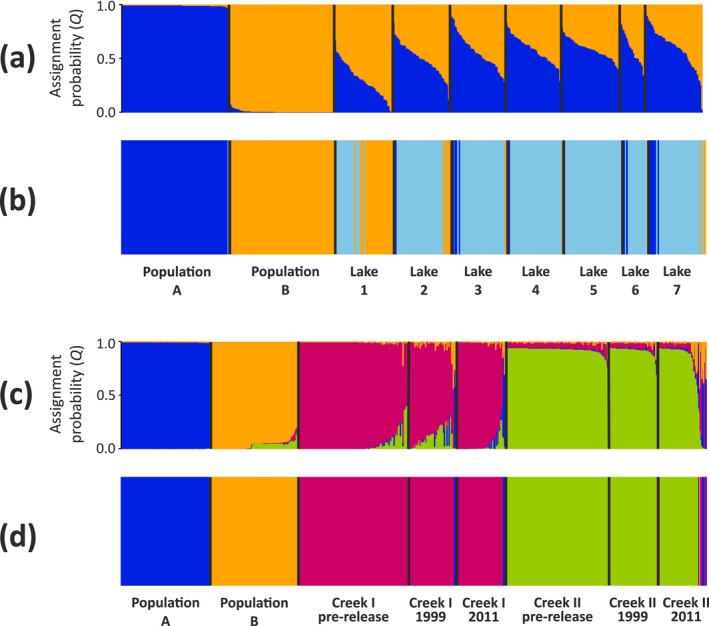
Results from analyses of population genetic structure in samples from the seven lakes above the waterfall (a, b) and the creek localities below the waterfall (c, d) using 96 SNPs. Assignment probabilities (*Q*) were obtained from structure and are shown in panels (a) and (c). Panels (b) and (d) depict the affiliation of each individual to a genetic cluster derived from the dapc analysis. Each fish is represented by one vertical bar in each panel.

**FIGURE 3:**
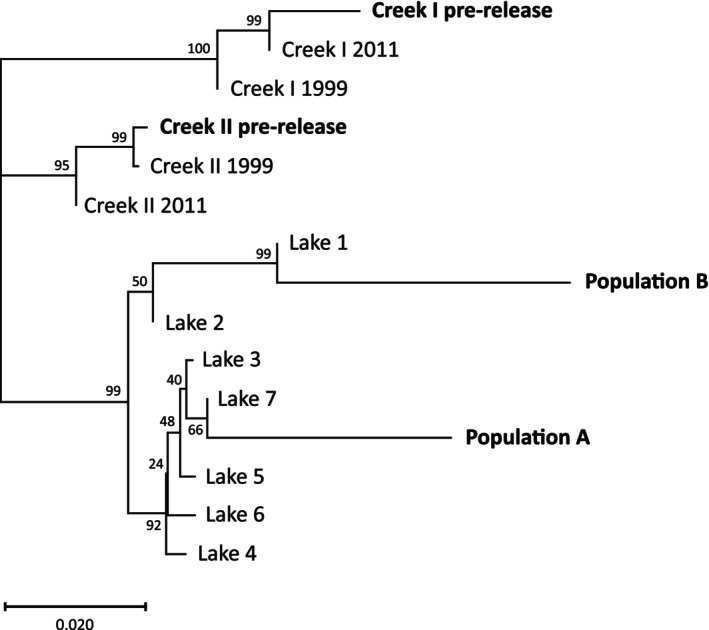
Phylogenetic tree illustrating the genetic relationships among the samples included in the present study. The tree was constructed using the neighbor‐joining method and is based on Nei's *D*_a_ distance. Numbers along the branches indicate bootstrap values in percentages.

**FIGURE 4:**
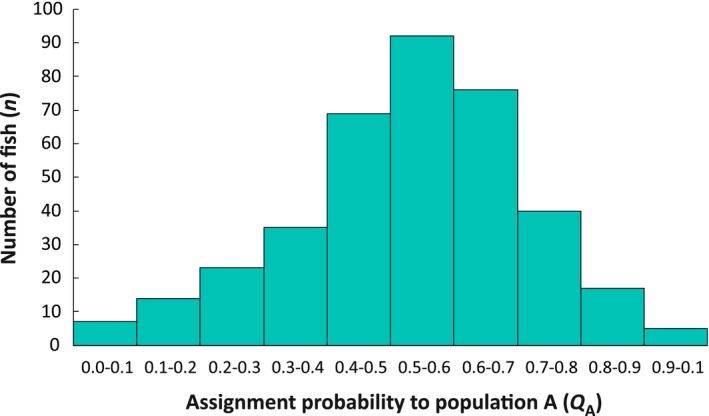
Distribution of assignment probabilities to population A (*Q*_A_) of the 378 fish from the seven lakes above the waterfall. *Q* values were obtained from structure using 96 SNPs.

There are significant pairwise genetic differences between all but two of the seven lakes above the waterfall (Lakes 3 and 7), with *F*_ST_ = 0.01–0.08 (Table S3). Further, structure identified *K* = 2 as the most likely number of genetic clusters above the waterfall. These clusters correspond to the released populations A and B and are present in all monitored lakes (Figure 2a), and hybridization is extensive (Figures 2a, 3, and 4). Few descendent fish (*n* = 4; c. 1%) can be considered as pure A or B (i.e., *Q* ≥ 0.99; Table S2). If we let the cut‐off drop to *Q* > 0.75, about 17% of the *n* = 378 fish can be considered as pure (*n* = 34 pure A and *n* = 31 pure B).

Our structure results are supported by the dapc analysis, which also identified the two clusters corresponding to populations A and B. In addition, dapc identified a third cluster (light blue in Figure 2b; Appendix S2) that contained most (81%) of the descendant fish above the waterfall, suggesting that hybridization is so extensive that dapc recognizes them as similar enough to constitute a single cluster. This observation is in line with the abovementioned low *F*_ST_ = 0.03 found among the seven lakes.

**FIGURE 5:**
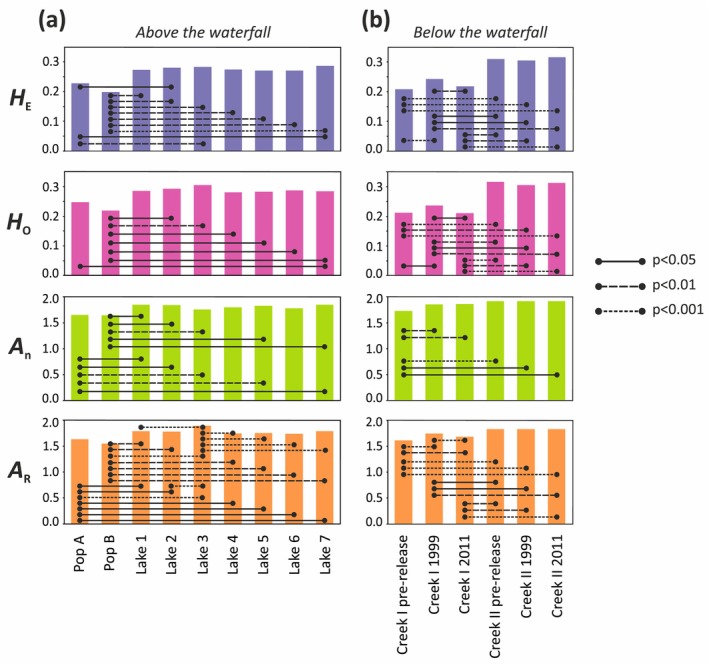
Measures of genetic diversity within introduced and native pre‐release populations and contemporary samples. Panel (a) shows introduced original populations A and B and the seven lakes, and panel (b) illustrates creek populations. Lines between bars indicate pairwise statistically significant divergence between the sampled populations. The significance level is indicated by the line structure (legend to the right in the figure).

Genetic diversity within each of the seven lakes is overall higher than in the two released populations A and B. Significant differences were observed between population B and all lakes with respect to both heterozygosity and allelic diversity, and between population A and all lakes regarding allelic diversity (Figure 5a; Table S4).

There is a consistent heterozygote excess (negative *F*_IS_ values) in all the lakes above the waterfall. This excess is only significant in one of the lakes (Lake 1), but the general tendency is significant when combining the information from all the lakes (Table 2). The estimates of effective size vary in the range *N*_eLD_ = 29–116 (Table 3). They are on the low side of estimates typically obtained for brown trout inhabiting similar environments in this area but not remarkably so (e.g., Jorde and Ryman 1996; Charlier et al. 2011; Andersson et al. 2022).

**TABLE 2:** *F* statistics with significance levels for the seven lakes above the waterfall and the creek localities below the waterfall.

Locality	*n*	*F*_IS_	*F*_IT_	*F*_ST_
Lake 1	60	−0.031***	**—**	**—**
Lake 2	60	−0.032	**—**	**—**
Lake 3	57	−0.067	**—**	**—**
Lake 4	57	−0.015	**—**	**—**
Lake 5	60	−0.036	**—**	**—**
Lake 6	26	−0.035	**—**	**—**
Lake 7	59	0.017	**—**	**—**
**Total (above the waterfall)**	**378**	**−0.027** ***	**0.006**	**0.032** ***
Creek I 1999	60	0.041***	**—**	**—**
Creek I 2011	60	0.054***	**—**	**—**
Creek II 1999	60	0.014	**—**	**—**
Creek II 2011	60	0.023	**—**	**—**

*Note:* Boldface indicates the "total" row for the material above the waterfall. The only bold values that are significant are FIS and FST, these are marked with "***" indicating *p* < 0.001.

**TABLE 3:** Effective population size above the waterfall estimated with the linkage disequilibrium method (*N*_eLD_) with 95% confidence intervals (CI).

Population/Lake	*N*_eLD_	95% CI
Population A	14	12–15
Population B	10	9–12
Lake 1	58	47–75
Lake 2	116	81–191
Lake 3	63	49–86
Lake 4	29	24–34
Lake 5	36	30–44
Lake 6	49	32–95
Lake 7	67	53–89

### 3.2 Genetic Effects on Phenotype

**FIGURE 6:**
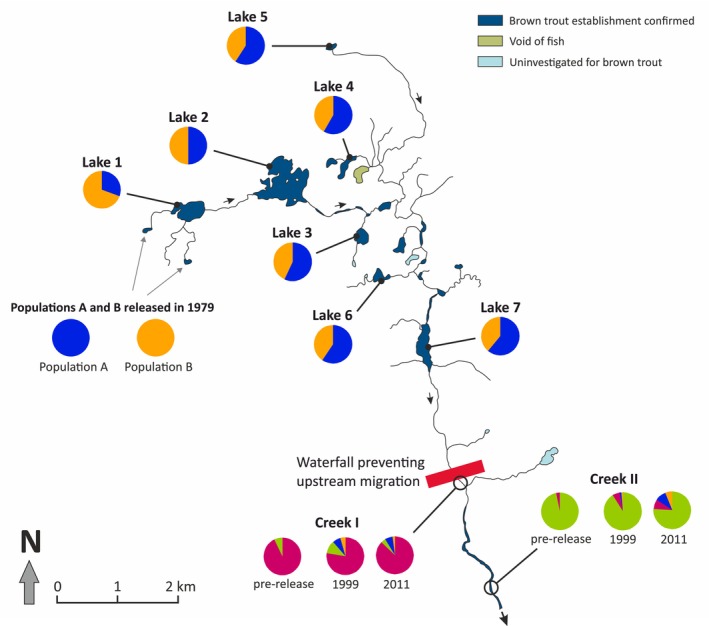
Genetic composition of established populations above the waterfall and native populations below the waterfall. Pie diagrams illustrate the proportion of genes originating from the released populations in the contemporary samples (expressed as average assignment to populations A and B above the waterfall, and to populations A, B, Creek I pre‐release and Creek II pre‐release below the waterfall). Black arrows show the direction of water flow.

Ancestry to populations A and B changes with distance from the release sites. Population B genes are most common in the lake closest to the release sites (Lake 1). In the next lake downstream (Lake 2) genes from A and B are equally common. Further downstream, population A genes dominate all lakes (Figure 6). This pattern is similar to that seen in the phylogenetic tree (Figure 3), where B is closer to Lake 2 than to any of the other lakes. In line with these observations, there is a significant positive correlation between the average assignment probability of a lake population (*Q*_A_) and the geographic distance from the release sites (Pearson's *r* = 0.89; *p* = 0.006; Figure S1).

For the analyses of body length and spawning frequency, we classified fish from the established populations into three genetic groups based on their assignment probability to population A (*Q*_A_). In total, 34 individuals were classified to group A (*Q*_A_ ≥ 0.75), 313 to group AB (*Q*_A_ = 0.26–0.74), and 31 to group B (*Q*_A_ ≤ 0.25).

Body length appeared to be affected by genetic group (*F* = 6.27; df = 2; *p* = 0.002), lake (*F* = 35.49; df = 6; *p* < 0.001), and age (*F* = 3093.86; df = 1; *p* < 0.001) when the 378 fish from the established populations were analyzed together with the 218 individuals from the originally released populations A and B (total *n* = 596; Table S5a). However, genetic group explained a relatively small part of the variation (only c. 2%), and when the original populations A and B were excluded, this effect disappeared altogether (Table S5b).

The proportion of mature individuals that would have spawned in the year of collection was much larger among fish belonging to the B group (35%) than in the A and AB groups (8% and 13%, respectively; Figure S2), and this difference was significant (*n* = 378; *χ* ^2^ = 11.03; df = 2; *p* = 0.004). However, the age distribution differed among genetic groups, with B being more skewed toward higher ages. Because of this, it is not clear if the difference in spawner frequency is attributed to genetics or simply an effect of the age structure within the sample.

### 3.3 Genetic Impact of Released Fish on Native Creek Populations

*K* = 4 was suggested as the most likely number of genetic clusters by both structure and dapc when the baselines for the released populations A and B and the two native, pre‐release creek populations from Creek I and Creek II were analyzed together with the 1999 and 2011 samples from the two creek localities. These clusters corresponded well to the four baseline populations (Figure 2c,d). The majority of fish in the post‐release samples in Creek I and Creek II (1999 and 2011) clustered with their respective pre‐release populations. There were, however, indications that migration from above the waterfall had occurred (Figures 2c,d and 6).

Immigrants from above the waterfall into the creek, i.e., individuals with an assignment probability *Q* < 0.25 to the creek location where they were caught, were observed in Creek I in both 1999 and 2011 (*n* = 4; i.e. 7% at both time points). In contrast, in Creek II, immigrants were only observed in 2011 (*n* = 5; 8%). Ten of these 13 immigrants appear to be hybrids between the introduced populations A and B, with a higher proportion of genes from population A (*Q*_A_ = 0.582–0.718). The remaining three carried genes almost exclusively from population A (*Q*_A_ = 0.793–0.994). We also observed downstream migration from Creek I to Creek II (i.e., immigrants were defined as fish caught in Creek II with an assignment probability *Q* ≥ 0.75 to Creek I; Table S6). Such immigrants occurred in Creek II in both 1999 (*n* = 1; 2%) and 2011 (*n* = 2; 3%).

Furthermore, we detected indications of introgression of genes from the introduced populations into the native ones at both localities at both points in time. Introgression was estimated as the proportion of genes originating from the released populations (i.e., average assignment probability to population A and B) in the creek sample after removing all individuals classified as immigrants. In Creek I, we found 5% introgression in 1999 and 3% in 2011, but this change was not statistically significant. For Creek II, introgression increased significantly from 3% in 1999 to 8% in 2011 (*p* = 0.02; Table S7). If also taking the immigrants into account, the total contribution to the gene pool of Creek I from the released populations is 11% and 9% in 1999 and 2011, respectively. For Creek II, the total proportion of alien genes was 3% and 16%, respectively (Figure 6).

### 3.4 Applying Indicators for Genetic Diversity Monitoring to Haravattsån

We applied indicators for monitoring genetic diversity to the populations in Creek I and Creek II. These indicators are used in national programs run by the Swedish Agency for Marine and Water Management and the Swedish Environmental Protection Agency (Johannesson and Laikre 2020, 2023; Andersson et al. 2022; Dussex et al. 2023; Kurland et al. 2024; Saha et al. 2024).

#### 3.4.1 Within‐Population Genetic Diversity Indicator ∆*H*

Overall, the pre‐release genetic variability was significantly higher in Creek II than in Creek I (Figure 5b), and this difference is consistent with the observations of Palm et al. (2003) who used allozyme data.

**FIGURE 7:**
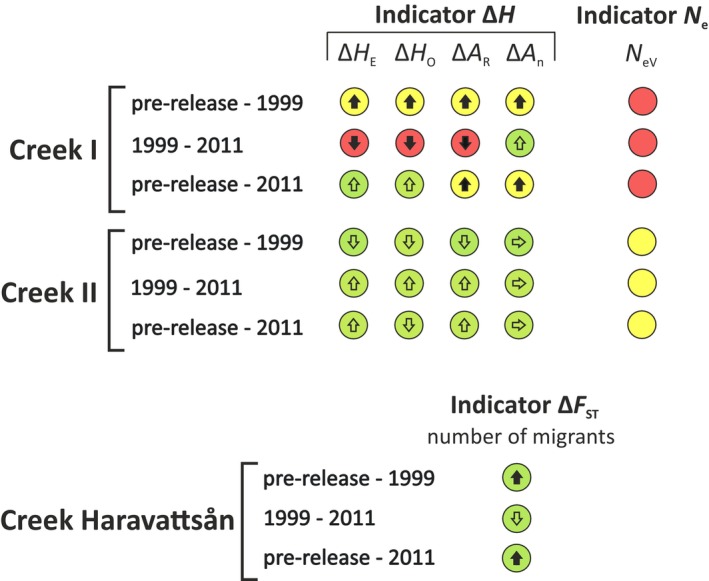
Indicators for genetic diversity applied to Creek I and Creek II. The colored circles show classification. Green = acceptable, yellow = warning, and red = alarm (cf. Andersson et al. 2022, their Figure 3). Arrows inside circles indicate the direction of change, with horizontal arrows meaning apparent stability (no change). Filled arrows indicate that the change is statistically significant (*p* < 0.05).

During the period of pre‐release to 1999, Creek I experienced a significant increase in all measures of genetic diversity (*H*_E_, *H*_O_, *A*_n_, and *A*_R_; Figures 5b and 7; Tables S4 and S8). The magnitude of change warrants a yellow/warning for the ∆*H* indicator (all diversity measures) due to the increase of genetic diversity governed by the introduction of non‐native genes. In contrast, we saw a significant decrease in *H*_E_, *H*_O_, and *A*_R_ from 1999 to 2011 in Creek I, which is so large that the indicator ∆*H* signals red alert (although the *A*_n_ change is green/acceptable; Figure 7; Tables S4 and S8). For the full monitoring period from pre‐release to 2011, a significant increase was observed only in allelic diversity (*A*_n_, and *A*_R_) resulting in warning signals (Figure 7; Tables S4 and S8). The creek locality further downstream, Creek II, experienced no statistically significant change in diversity during the monitoring period, warranting the status green/acceptable for indicator ∆*H* (Figure 7).

#### 3.4.2 *N*_e_ Indicator

Effective population size (*N*_e_) was estimated for the time periods pre‐release‐1999, 1999–2011, and pre‐release‐2011 for both creek localities, and most estimates were rather small. In Creek I, all estimates were below 50, whereas those for Creek II were somewhat higher (*N*_eV_ = 62–109; *N*_eLD_ = 65–124; Tables 4 and S9).

**TABLE 4:** Effective population size for the populations Creek I and Creek II estimated using the temporal (*N*_eV_) and the linkage disequilibrium (*N*_eLD_) methods, with 95% CIs for *N*_eV_.

Population	Time period	*N*_eV_	95% CI (*N*_eV_)	*N*_eLD_ (harmonic mean)
Creek I	Pre‐release – 1999	12	10–17	7
	1999–2011	18	14–26	6
	Pre‐release – 2011	23	17–34	13
Creek II	Pre‐release – 1999	109	58–804	124
	1999–2011	62	39–153	65
	Pre‐release – 2011	79	56–134	72

*Note:* *N*_eLD_ is the harmonic mean over *N*_eLD_ at the two points in time shown in the column “Time period”.

In local populations belonging to a population system, both *N*_eV_ and *N*_eLD_ tend to underestimate inbreeding effective size (*N*_eI_), which is the effective size referred to in Franklin's (1980) 50/500 rule (Ryman et al. 2019, 2023). Thus, we consistently used the largest of *N*_eV_ and *N*_eLD_ as an estimate for the *N*_e_ indicator. This resulted in red/alarm for Creek I for all three time periods. In Creek II, the *N*_e_ indicator is yellow/warning for all periods (Figure 7; Table S9).

#### 3.4.3 Between‐Population Genetic Diversity Indicator ∆ *F*_ST_

The ∆*F*_ST_ indicator, which measures temporal change in genetic divergence between populations, is green/acceptable for all comparisons (Figure 7). Δ*F*_ST_ suggests that genetic divergence between the two creek localities has decreased during the period of pre‐release to 1999, from *F*_ST_ = 0.21 to *F*_ST_ = 0.16, indicating that migration has increased over this period (Table S10). However, this change does not exceed the currently applied threshold corresponding to a 50% gene flow increase for the yellow classification, and thus green/acceptable status applies for this period (Figure 7). *F*_ST_ increased from 0.16 to 0.18 between 1999 and 2011, but this change is not statistically significant (Table S10). Applying the Δ*F*_ST_ indicator for the full period from before the release to 2011 gives a statistically significant reduction of *F*_ST_ but within what is classified as safe limits (Figure 7; Table S10).

### 3.5 SNPs and Allozymes Provide Similar Pictures of Genetic Structure

We find a high degree of similarity between the SNP and the allozyme data. There is a strong, significant positive correlation between the within‐lake average assignment probability to population B (*Q*_B_) and the frequency of the *50* allele at the *G3PDH‐2* locus that was fixed in population B (Pearson's *r* = 0.92; *p* = 0.003; Figure S3). Thus, the allele frequencies at *G3PDH‐2* in the seven lakes show the same pattern as the average assignment probabilities to populations A and B (Figure S4). Further, when monitoring the *G3PDH‐2* allele frequencies in Lakes 1, 2, and 7 using an extended allozyme dataset (*n* = 11,767; Table S11), we observe similar patterns as in the fish scored for SNPs in these lakes (Figure S5).

With respect to the creek localities, the *G3PDH‐2 50* allele appears in Creek I for the first time in 1988 (Creek I was fixed for the *100* allele prior to receiving immigrants; Palm et al. 2003), suggesting that immigration started to occur around that time. After 1988, this allele occurs at a frequency of about 2%–10% in this locality (Figure S5). The pre‐release population in Creek II was polymorphic at the *G3PDH‐2* locus, with the *50* allele naturally present prior to the release of populations A and B.

## 4 Discussion

We identified two genetic clusters that correspond to the released populations A and B in all seven lakes above the waterfall. We found extensive hybridization between populations A and B within all lakes, with small but significant genetic divergence among localities. Our results suggest that immigration and introgression into the native populations occur from fish migrating over the waterfall. Our major findings are:

1. Hybridization has resulted in considerably higher genetic diversity in established populations than in the released ones A and B.
2. Differences in migratory behavior between the original populations in their native environments remain to some degree in the new environment. Genes from the more resident population (B) dominate in the lake closest to the site of release, whereas genes from the more migratory population (A) are most common further downstream.
3. Early maturity is more common among descendants from the more resident population B. Fish in the genetic group AB (i.e., fish with 0.25 ≤ *Q*_A_ ≤ 0.75) appear to exhibit late maturation similar to that of population A.
4. Genes from the released populations have spread to the native creek populations downstream of a waterfall, but introgression is much smaller than that between the released ones above the waterfall.
5. Recently adopted indicators for genetic diversity signaled a change in diversity (indicator ∆*H*) in one of the two monitored creek populations when applying current threshold values.
6. The results from the 96 SNPs applied in this study show high consistency with those based on allozymes used in the early phases of the project.

### 4.1 Hybridization Above the Waterfall

Palm and Ryman (1999) reported a lower frequency of hybrids in the F1 generation than expected under random mating. In our present data representing generations 5–6, we find that extensive hybridization between the released populations A and B has occurred. Further, the present populations above the waterfall have significantly higher levels of genetic diversity than the released ones (Figure 5a; Table S4). There is also a consistent excess of heterozygotes (negative *F*_IS_ values) above the waterfall that is significant when combining the information from all lakes (Table 2); such an excess is in line with expectations during a phase of genetic homogenization through continued hybridization. Taken together, these findings suggest that the released fish had a tendency to mate within their own population in the first few generations but that this trend has become weaker over time.

Hybridization between genetically distinct populations has been shown to increase fitness in a novel environment in organisms such as cichlid fishes (Meier et al. 2019), sterlets (Shivaramu et al. 2020), sunflowers (Mitchell et al. 2019), yeast (Zhang et al. 2020), and salmonid fishes (Fukui and Koizumi 2019). In the present case, however, it is unclear whether the hybridization between the two released populations has been beneficial for the successful establishment of these populations over only a few generations or just the result of limited barriers to gene flow.

### 4.2 Genetic–Phenotypic Correlations

Body size differences between the released populations A and B in their original environments disappear in the new environment with subsequent hybridization. The small differences in body length observed between genetic groups are only significant when including the baseline populations in the analysis, but even then, the genetic group only explained about 2% of the total variation (Table S5a,b). This result is in line with the observations of Palm and Ryman (1999) who present similar effects of genetic background for the first two generations after the release. Our observations indicate a high degree of plasticity for body size, which has been shown experimentally in salmonid fishes (Oomen and Hutchings 2015). In the closely related Atlantic salmon, body size can change dramatically in response to environmental variation (Debes et al. 2014) or as a result of hybridization between different populations (Morris et al. 2011).

We find an association between genetic contribution from released populations A and B and the migration pattern (Figure S1), something that was also observed by Palm and Ryman (1999), suggesting that this trait is heritable. This association with migration distance represents the most striking correlation between genetic background and a phenotypic characteristic we observe in the present data. The possible genetic basis for this relationship cannot be determined from this study, but the clear correlation over a wide range of *Q* values (Figures 6 and S1) indicates some form of polygenic inheritance, possibly with contribution from environmental factors. This finding suggests that some phenotypic characteristics, such as migratory behavior, need to be taken into account when planning for the intentional translocation of brown trout populations.

### 4.3 Introgression Into the Native Creek Populations

Introgression rates into the natural populations below the waterfall suggest that hybridization with immigrants was considerably lower than that between A and B above the waterfall. *F*_IS_ values are consistently positive below the waterfall and highly significant in the upper creek locality (Creek I), suggesting sampling from a mixture of genetically distinct populations, i.e., a Wahlund effect (Table 2).

In Creek I, we observed four immigrants among the 60 individuals (7%) in each of the 1999 and 2011 samples. At the same time, there is a small but nonsignificant decrease in introgression over this period, from 5% in 1999 to 3% in 2011. In the location further downstream, Creek II, we identify no immigrants and only weak indications of introgression in 1999 (3%). In contrast, in the 2011 sample, we find five immigrants from the released descendants (8%) and an introgression of c. 8%. Thus, it seems that Creek II was affected by the release somewhat later than Creek I.

Clearly, monitoring over an extended period of time may be necessary for detecting the effects of a release. In the present case, for example, the 1999 sampling in the creek took place 20 years after the release, and the second one occurred 12 years later. In 1999, we got the impression that immigration and introgression were mainly confined to the upper creek location (Creek I), whereas the lower one (Creek II) seemed rather unaffected. However, 12 years later, the picture has changed, and Creek II appears to have more introgression than the upper location. Without the sample from 2011 (32 years after the release), we could have stayed with the erroneous impression that introgression from the release into natural populations was not a major issue in the creek as a whole. Obviously, it can be very difficult to predict the long‐term impact on biodiversity from intentional and unintentional releases, even if implementing a fairly ambitious genetic monitoring program to check for such effects. In practice, it may not even be possible to perform an investigation several decades after the release event, implying that the need for translocations should be evaluated with considerable care.

### 4.4 Indicators for Genetic Diversity

The indicators suggest a genetic change in the creek. However, without the information that fish had been released in the area, it would not have been perfectly clear how to interpret the increase of all measures of the Δ*H* indicator in Creek I. We observe a significant increase in genetic diversity from pre‐release until 1999 of a magnitude resulting in warning/yellow (Figures 5b and 7), which is then followed by a reduction in the next time interval (1999–2011) resulting in alarm/red. There is an overall increase over the entire period (pre‐release‐2011). Had we not known about the release, it would have been difficult to interpret these observations. Thus, under some circumstances, the traffic light system may not be sufficient by itself for making informed decisions on a proper management strategy.

In contrast to Creek I, we see no statistically significant change in genetic diversity in the population further downstream (Creek II) over the monitoring period. This observation may either reflect a true lack of change or be due to statistical difficulties in detecting a change. With respect to statistics, the pre‐release level of genetic diversity was higher in Creek II, making it more difficult to notice a minor diversity shift. Further, some alleles that are “diagnostic” for the released fish occurred naturally in Creek II prior to the release, potentially masking the effect of immigration and introgression.

The indicator for between‐population genetic diversity (Δ*F*_ST_) detects a significant decrease in *F*_ST_ between the two creek localities during pre‐release to 1999 as well as during the full period (pre‐release to 2011), indicating increasing gene flow between the creek localities. However, the change of *F*_ST_ does not result in warning signals with currently applied threshold values. The threshold for “warning” requires a reduction of *F*_ST_ reflecting a 50% increase in the number of migrants (cf. Andersson et al. 2022). The observation of a decreasing *F*_ST_ that is not captured by the Δ*F*_ST_ indicator, as well as similar observations in Atlantic salmon (Johannesson and Laikre 2023; Andersson et al. unpublished data), suggests that adjustments of the threshold values might be warranted for this indicator. In the case of Creek Haravattsån, the threshold for maximum increase of genetic exchange would need to be lowered from 50% to 40% in order for the Δ*F*_ST_ indicator to result in a warning signal.

So far, these indicators have only been applied to a few species (Atlantic salmon, Johannesson and Laikre 2023; brown trout, Andersson et al. 2022; Kurland et al. 2024; moose ( *Alces alces* ), Dussex et al. 2023; and Arctic char, Saha et al. 2024). Adjustments and modifications of the indicators are justified following the insights gained from these initial applications, including the present one.

### 4.5 Comparing Molecular Markers

The currently observed dispersal pattern, where genes from population A have spread further downstream than genes from population B, was indicated already in the first generation (F1) of fish established in the two uppermost lakes (Lakes 1 and 2), as studied with allozymes (Palm and Ryman 1999). This dispersal pattern is also supported by whole genome sequencing (WGS) using data from Lakes 1 and 7 (Kurland et al. 2022). Our present study demonstrates that this pattern occurs in the entire water system where brown trout is now established (Figures 2a,b and 6).

We find a strong correlation between the frequency of the *50*‐allele at the *G3PDH‐2* allozyme locus (marker for population B when released in 1979) and assignment probability to B with the presently applied SNP array. This indicates that in some cases older marker systems with few loci may be helpful to provide an adequate picture of population genetic structure and dynamics (e.g., Saha et al. 2022; cf. results from Wennerström et al. 2016 with Dussex et al. 2023). It is important to acknowledge the utility of older molecular markers for monitoring (Bertola et al. 2023). The extensive data already available from such techniques, and the relative ease of using them may facilitate improved genetic monitoring, which could help reduce the conservation genetics gap (Taylor et al. 2017; Klütsch and Laikre 2021).

In other scenarios, genomics provides an important avenue to further our understanding of successful establishment in novel environments, e.g., to track which genes or parts of genomes and phenotypes are successful, which parts of the genome are particularly susceptible to hybridization, or to identify emerging local adaptations and to distinguish these from plasticity. For example, in the brown trout of the present water system, a tendency for different selective pressures acting in the top lake (Lake 1) compared to the one further downstream (Lake 7) has been observed (Kurland et al. 2022).

### 4.6 Ecological Implications of the Release

The most obvious ecological effect of the release is that the translocated fish have reproduced successfully, spread throughout the water system above the waterfall, and to a considerable extent replaced the Arctic char, which originally was the only fish present. In addition, the introduction of trout appears to be coupled to a selective sweep in the Arctic char, as shown by Saha et al. (2024), but not in reduction of genetic diversity.

In spite of their different origins and possible local adaptations, both the released populations have spread their genes to a similar extent in most of the lakes. Positive effects of hybridization between A and B may have facilitated this spread of genes from both populations, but the lack of local conspecifics above the waterfall may also have contributed to the success.

The effective size is rather small in the lakes as well as in the released populations (Table 3), but this appears not to have affected the spread of the introduced populations. From a theoretical perspective, this is not surprising, however. First, the bottlenecks of A and B only refer to a single generation where loss of heterozygosity is 1/2*N*_e_. Thus, with an *N*_e_ of, say, *N*_e_ = 10, only 5% is lost and 95% is retained. Second, in the following generations, the heterozygosity loss is determined by the effective size of the metapopulation above the waterfall rather than the local effective size of the particular subpopulations, and this global effective size is typically much larger than the local ones. Finally, in subdivided populations, the linkage disequilibrium effective size (*N*_eLD_) tends to systematically underestimate the inbreeding effective size (*N*_eI_) which is the proper effective size for predicting loss of heterozygosity (Ryman et al. 2019).

For the natural populations below the waterfall, the most striking observation refers to the seemingly low rates of introgression from the introduced populations. Hybridization between A and B is extensive above the waterfall, but so far, these populations do not appear to hybridize readily with the native ones below the waterfall. The reason for this difference is not clear, but may be due to competition with natural trout in the creek. Alternatively, the A and B populations may not be sufficiently adapted to the specific conditions of a creek environment. We also note that the number of trout migrating over the waterfall appears fairly small, and the relatively low introgression rate in the creek may basically reflect that only a minor proportion of the matings would result in hybridization also under more or less panmictic conditions.

We anticipate that descendants of the introduced trout will continue to trickle down over the waterfall, potentially resulting in further introgression. It may be counteracted by natural selection in the creek. Alternatively, natural selection above the waterfall will make the fish better adapted to the general conditions of the overall subdrainage, resulting in more competitive immigrants into the creek and increased introgression. The outcome can only be addressed through continued monitoring.

## Conflicts of Interest

The authors declare no conflicts of interest.

## Benefit Sharing Statement

This research is closely linked to Sweden's national environmental monitoring of genetic diversity run by the Swedish Agency for Marine and Water Management (SwAM). The indicators for genetic diversity applied in this study were developed in ongoing science–management–policy implementation work, and the brown trout is one of the focal species of the monitoring of genetic diversity in aquatic systems. This study contributes to the evaluation and adjustment of these indicators using empirical data, with results being shared with agencies and the broader scientific community, both nationally and internationally.

## Supporting information


**Supinfo S1**.

**Supinfo S2**.

## Data Availability

All measurements underlying the genetic indicator classifications are provided in the Supporting Information (Tables S8–S10). Individual genotype data are available at Dryad (https://doi.org/10.5061/dryad.37pvmcvvq).

## References

[eva70084-bib-0001] Allendorf, F. W. , R. F. Leary , P. Spruell , and J. K. Wenburg . 2001. “The Problems With Hybrids: Setting Conservation Guidelines.” Trends in Ecology & Evolution 16: 613–622. 10.1016/S0169-5347(01)02290-X.

[eva70084-bib-0071] Allendorf, F. W. , and N. Ryman . 2002. “The Role of Genetics in Population Viability Analysis.” In Population Viability Analysis, edited by S. R. Beissinger and D. R. McCullough , 50–85. University of Chicago Press.

[eva70084-bib-0002] Andersson, A. , S. Karlsson , N. Ryman , and L. Laikre . 2022. “Monitoring Genetic Diversity With New Indicators Applied to an Alpine Freshwater Top Predator.” Molecular Ecology 31: 6422–6439. 10.1111/mec.16710. 36170147 PMC10091952

[eva70084-bib-0003] Bertola, L. D. , A. Brüniche‐Olsen , F. Kershaw , et al. 2023. “A Pragmatic Approach for Integrating Molecular Tools Into Biodiversity Conservation.” Conservation Science and Practice 6: e13053. 10.1111/csp2.130531.

[eva70084-bib-0004] Carvalho, S. B. , J. Torres , P. Tarroso , and G. Velo‐Antón . 2019. “Genes on the Edge: A Framework to Detect Genetic Diversity Imperiled by Climate Change.” Global Change Biology 25: 4034–4047. 10.1111/gcb.14740. 31230387

[eva70084-bib-0005] Ceballos, G. , P. R. Ehrlich , and R. Dirzo . 2017. “Biological Annihilation via the Ongoing Sixth Mass Extinction Signaled by Vertebrate Population Losses and Declines.” PNAS 114: E6089–E6096. 10.1073/pnas.1704949114. 28696295 PMC5544311

[eva70084-bib-0006] Charlier, J. , A. Palmé , L. Laikre , J. Andersson , and N. Ryman . 2011. “Census (NC) and Genetically Effective (Ne) Population Size in a Lake‐Resident Population of Brown Trout *Salmo trutta* .” Journal of Fish Biology 79: 2074–2082. 22141907 10.1111/j.1095-8649.2011.03124.x

[eva70084-bib-0007] Debes, P. V. , D. J. Fraser , M. Yates , and J. A. Hutchings . 2014. “The Between‐Population Genetic Architecture of Growth, Maturation, and Plasticity in Atlantic Salmon.” Genetics 196: 1277–1291. 24473933 10.1534/genetics.114.161729 PMC3982675

[eva70084-bib-0008] Do, C. , R. S. Waples , D. Peel , G. M. Macbeth , B. J. Tillett , and J. R. Ovenden . 2014. “NeEstimator V2: Re‐Implementation of Software for the Estimation of Contemporary Effective Population Size (Ne) From Genetic Data.” Molecular Ecology Resources 14: 209–214. 23992227 10.1111/1755-0998.12157

[eva70084-bib-0009] Dupoué, A. , A. Trochet , M. Richard , et al. 2020. “Genetic and Demographic Trends From Rear to Leading Edge Are Explained by Climate and Forest Cover in a Cold‐Adapted Ectotherm.” Diversity and Distributions 27: 267–281. 10.1111/ddi.13202.

[eva70084-bib-0010] Dussex, N. , S. Kurland , R.‐A. Olsen , et al. 2023. “Range‐Wide and Temporal Genomic Analyses Reveal the Consequences of Near‐Extinction in Swedish Moose.” Communications Biology 6: 1035. 10.1038/s42003-023-05385-x. 37848497 PMC10582009

[eva70084-bib-0011] Falush, D. , M. Stephens , and J. K. Pritchard . 2003. “Inference of Population Structure Using Multilocus Genotype Data: Linked Loci and Correlated Allele Frequencies.” Genetics 164: 1567–1587. 12930761 10.1093/genetics/164.4.1567 PMC1462648

[eva70084-bib-0012] Filipsson, O. 1994. “New Fish Populations Through Transplantation or Release of Fish (In Swedish).” Information från Sötvattenslaboratoriet 2: 1–65.

[eva70084-bib-0013] Frank, B. M. , J. J. Piccolo , and P. V. Baret . 2011. “A Review of Ecological Models for Brown Trout: Towards a New Demogenetic Model.” Ecology of Freshwater Fish 20: 167–198.

[eva70084-bib-0070] Franklin, I. R. 1980. “Evolutionary Changes in Small Populations.” In Conservation Biology: an Evolutionary‐Ecological Perspective, edited by M. E. Soulé and B. A. Wilcox , 135–139. Sinauer Associates Inc.

[eva70084-bib-0014] Fukui, S. , and I. Koizumi . 2019. “Hybrids as Potential Mediators Spreading Non‐Native Genes: Comparison of Survival, Growth, and Movement Among Native, Introduced and Their Hybrid Salmonids.” Ecology of Freshwater Fish 29: 280–288. 10.1111/eff.12513.

[eva70084-bib-0015] Gordeeva, N. V. , and E. A. Salmenkova . 2011. “Experimental Microevolution: Transplantation of Pink Salmon Into the European North.” Evolutionary Ecology 25: 657–679. 10.1007/s10682-011-9466-x.

[eva70084-bib-0016] Goudet, J. 2003. Fstat (ver. 2.9.4), a Program to Estimate and Test Population Genetics Parameters . http://www.unil.ch/izea/softwares/fstat.html.

[eva70084-bib-0017] Hampe, A. , M.‐H. Pemonge , and R. J. Petit . 2013. “Efficient Mitigation of Founder Effects During the Establishment of a Leading‐Edge Oak Population.” Proceedings of the Royal Society B 280: 20131070. 10.1098/rspb.2013.1070. 23782887 PMC3712427

[eva70084-bib-0018] Hesthagen, T. , and O. T. Sandlund . 2004. “Fish Distribution in a Mountain Area in South‐Eastern Norway: Human Introductions Overrule Natural Immigration.” Hydrobiologia 521: 49–59.

[eva70084-bib-0019] Johannesson, K. , and L. Laikre . 2020. “Monitoring of Genetic Diversity in Environmental Monitoring (in Swedish).” Report to the Swedish Agency for Marine and Water Management (dnr. HaV 3642‐2018, 3643‐2018).

[eva70084-bib-0020] Johannesson, K. , and L. Laikre . 2023. “Environmental Monitoring of Genetic Diversity (in Swedish).” Report to the Swedish Agency for Marine and Water Management (dnr. HaV 1716‐22, 2007–21, 1717).

[eva70084-bib-0021] Jombart, T. 2008. “Adegenet: A R Package for the Multivariate Analysis of Genetic Markers.” Bioinformatics 24: 1403–1405. 10.1093/bioinformatics/btn129. 18397895

[eva70084-bib-0022] Jombart, T. , and I. Ahmed . 2011. “Adegenet 1.3‐1: New Tools for the Analysis of Genome‐Wide SNP Data.” Bioinformatics 27: 3070–3071. 10.1093/bioinformatics/btr521. 21926124 PMC3198581

[eva70084-bib-0023] Jombart, T. , S. Devillard , and F. Balloux . 2010. “Discriminant Analysis of Principal Components: A New Method for the Analysis of Genetically Structured Populations.” BMC Genetics 11: 94. 10.1186/1471-2156-11-94. 20950446 PMC2973851

[eva70084-bib-0024] Jonsson, B. , and N. Jonsson . 2011. “Species Diversity.” In Ecology of Atlantic Salmon and Brown Trout, edited by B. Jonsson and N. Jonsson , 23–66. Springer.

[eva70084-bib-0025] Jorde, P. E. , and N. Ryman . 1996. “Demographic Genetics of Brown Trout (*Salmo trutta*) and Estimation of Effective Population Size From Temporal Change of Allele Frequencies.” Genetics 143: 1369–1381. 8807308 10.1093/genetics/143.3.1369 PMC1207405

[eva70084-bib-0026] Jorde, P. E. , and N. Ryman . 2007. “Unbiased Estimator for Genetic Drift and Effective Population Size.” Genetics 177: 927–935. 17720927 10.1534/genetics.107.075481 PMC2034655

[eva70084-bib-0027] Karlsson, S. , O. H. Diserud , P. Fiske , and K. Hindar . 2016. “Widespread Genetic Introgression of Escaped Farmed Atlantic Salmon in Wild Salmon Populations.” ICES Journal of Marine Science 73: 2488–2498.

[eva70084-bib-0028] Klütsch, C. F. C. , and L. Laikre . 2021. “Closing the Conservation Genetics Gap: Integrating Genetic Knowledge in Conservation Management to Ensure Evolutionary Potential.” In Closing the Knowledge‐Implementation Gap in Conservation Science. Wildlife Research Monographs, edited by C. C. Ferreira and C. F. C. Klütsch , vol. 4. Springer. 10.1007/978-3-030-81085-6_3.

[eva70084-bib-0029] Kurland, S. , N. Rafati , N. Ryman , and L. Laikre . 2022. “Genomic Dynamics of Brown Trout Released to a Novel Environment.” Ecology and Evolution 12: e9050. 10.1002/ece3.9050. 35813906 PMC9251865

[eva70084-bib-0030] Kurland, S. , A. Saha , N. Keehnen , et al. 2024. “New Indicators for Monitoring Genetic Diversity Applied to Alpine Brown Trout Populations Using Whole Genome Sequence Data.” Molecular Ecology 33: e17213. 10.1111/mec.17213. 38014725

[eva70084-bib-0031] Laikre, L. , A. Palmé , M. Joseffson , F. Utter , and N. Ryman . 2006. “Release of Alien Populations in Sweden.” Ambio 35, no. 5: 255–261. 10.1579/05-a-060r.1. 16989510

[eva70084-bib-0032] Laikre, L. , M. K. Schwartz , R. S. Waples , N. Ryman , and The GeM Working Group . 2010. “Compromising Genetic Diversity in the Wild: Unmonitored Large‐Scale Release of Plants and Animals.” Trends in Ecology & Evolution 25: 520–529. 10.1016/j.tree.2010.06.013. 20688414

[eva70084-bib-0033] Marco‐Rius, F. , G. Sotelo , P. Caballero , and P. Moran . 2013. “Insights for Planning an Effective Stocking Program in Anadromous Brown Trout (*Salmo trutta*).” Canadian Journal of Fisheries and Aquatic Sciences 70: 1092–1100.

[eva70084-bib-0034] Meier, J. I. , R. B. Stelkens , D. A. Joyce , et al. 2019. “The Coincidence of Ecological Opportunity With Hybridization Explains Rapid Adaptive Radiation in Lake Mweru Cichlid Fishes.” Nature Communications 10: 5391. 10.1038/s41467-019-13278-z. PMC6890737 31796733

[eva70084-bib-0035] Mitchell, N. , G. L. Owens , S. M. Hovick , L. H. Rieseberg , and K. D. Whitney . 2019. “Hybridization Speeds Adaptive Evolution in an Eight‐Year Field Experiment.” Scientific Reports 9: 6746. 10.1038/s41598-019-43119-4. 31043692 PMC6494830

[eva70084-bib-0036] Morris, M. R. , D. J. Fraser , J. Eddington , and J. A. Hutchings . 2011. “Hybridization Effects on Phenotypic Plasticity: Experimental Compensatory Growth in Farmed‐Wild Atlantic Salmon.” Evolutionary Applications 4: 444–458. 25567994 10.1111/j.1752-4571.2010.00159.x PMC3352526

[eva70084-bib-0037] Muhlfeld, C. C. , D. C. Dauwalter , V. S. D'Angelo , et al. 2019. “Global Status of Trout and Char: Conservation Challenges in the Twenty‐First Century.” In Book: Trout and Char of the World. American Fisheries Society.

[eva70084-bib-0038] Muhlfeld, C. C. , S. T. Kalinowski , T. E. McMahon , et al. 2009. “Hybridization Rapidly Reduces Fitness of a Native Trout in the Wild.” Biology Letters 5: 328–331. 10.1098/rsbl.2009.0033. 19324629 PMC2679930

[eva70084-bib-0039] Nei, M. 1987. Molecular Evolutionary Genetics, 512. Columbia University Press.

[eva70084-bib-0040] Oomen, R. A. , and J. A. Hutchings . 2015. “Genetic Variability in Reaction Norms in Fishes.” Environmental Reviews 23: 353–366.

[eva70084-bib-0041] Palm, S. , S. Karlsson , and O. H. Diserud . 2021. “Genetic Evidence of Farmed Straying and Introgression in Swedish Wild Salmon Populations.” Aquaculture Environment Interactions 13: 505–513.

[eva70084-bib-0042] Palm, S. , L. Laikre , P. E. Jorde , and N. Ryman . 2003. “Effective Population Size and Temporal Genetic Change in Stream Resident Brown Trout (*Salmo trutta*, L.).” Conservation Genetics 4: 249–264.

[eva70084-bib-0043] Palm, S. , and N. Ryman . 1999. “Genetic Basis of Phenotypic Differences Between Transplanted Stocks of Brown Trout.” Ecology of Freshwater Fish 8: 169–180.

[eva70084-bib-0044] Peakall, R. , and P. E. Smouse . 2006. “GenAlEx 6: Genetic Analysis in Excel. Population Genetic Software for Teaching and Research.” Molecular Ecology Notes 6: 288–295. 10.1093/bioinformatics/bts460 PMC3463245 22820204

[eva70084-bib-0045] Peakall, R. , and P. E. Smouse . 2012. “GenAlEx 6.5: Genetic Analysis in Excel. Population Genetic Software for Teaching and Research – An Update.” Bioinformatics 28: 2537–2539. 22820204 10.1093/bioinformatics/bts460 PMC3463245

[eva70084-bib-0046] Pearman, P. B. , O. Broennimann , T. Albayrak , et al. 2024. “Monitoring of Species' Genetic Diversity in Europe Varies Greatly and Overlooks Potential Climate Change Impacts.” Nature Ecology & Evolution 8, no. 2: 267–281. 10.1038/s41559-023-02260-0. 38225425 PMC10857941

[eva70084-bib-0047] Pritchard, J. K. , M. Stephens , and P. Donnelly . 2000. “Inference of Population Structure Using Multilocus Genotype Data.” Genetics 155: 945–959. 10835412 10.1093/genetics/155.2.945 PMC1461096

[eva70084-bib-0048] R Core Team . 2021. R: A Language and Environment for Statistical Computing. R Foundation for Statistical Computing. https://www.R‐project.org/.

[eva70084-bib-0049] Raymond, M. , and F. Rousset . 1995. “GENEPOP (Version 1.2): Population Genetics Software for Exact Tests and Ecumenicism.” Journal of Heredity 86: 248–249.

[eva70084-bib-0050] Rousset, F. 2008. “GENEPOP'007: A Complete Reimplementation of the Genepop Software for Windows and Linux.” Molecular Ecology Resources 8: 103–106. 21585727 10.1111/j.1471-8286.2007.01931.x

[eva70084-bib-0051] Ryman, N. 1983. “Patterns of Distribution of Biochemical Genetic Variation in Salmonids: Differences Between Species.” Aquaculture 33: 1–21.

[eva70084-bib-0052] Ryman, N. , L. Laikre , and O. Hössjer . 2019. “Do Estimates of Contemporary Effective Population Size Tell Us What We Want to Know?” Molecular Ecology 28: 1904–1918. 10.1111/mec.15027. 30663828 PMC6850010

[eva70084-bib-0053] Ryman, N. , L. Laikre , and O. Hössjer . 2023. “Variance Effective Population Size Is Affected by Census Size in Sub‐Structured Populations.” Molecular Ecology Resources 23: 1334–1347. 37122118 10.1111/1755-0998.13804

[eva70084-bib-0054] Ryman, N. , R. Öhman , G. Ståhl , A. Nilsson , U. Lagercrantz , and A. Herlitz . 1986. Genetisk variation hos laxartad fisk – en snabbt försvinnande naturresurs: information om genetisk forskning i Jämtland. Division of Genetics, Stockholm University. Lantbruksnämnden Jämtlands län.

[eva70084-bib-0055] Saha, A. , A. Andersson , S. Kurland , et al. 2022. “Whole‐Genome Resequencing Confirms Reproductive Isolation Between Sympatric Demes of Brown Trout (*Salmo trutta*) Detected With Allozymes.” Molecular Ecology 33: 1–14. 10.1111/mec.16252. 34699656

[eva70084-bib-0056] Saha, A. , S. Kurland , V. E. Kutschera , et al. 2024. “Monitoring Genome‐Wide Diversity Over Contemporary Time With New Indicators Applied to Arctic Charr Populations.” Conservation Genetics 25: 513–531. 10.1007/s10592-023-01586-3.

[eva70084-bib-0057] Shivaramu, S. , I. Lebeda , V. Kašpar , and M. Flajšhans . 2020. “Intraspecific Hybrids Versus Purebered: A Study of Hatchery‐Reared Populations of Sterlet *Acipenser ruthenus* .” Animals 10: 1149. 10.3390/ani10071149. 32645877 PMC7401548

[eva70084-bib-0058] StatSoft, Inc . 2005. “STATISTICA (data analysis software system) version 7.1.” www.statsoft.com.

[eva70084-bib-0059] Takezaki, N. , M. Nei , and K. Tamura . 2010. “POPTREE2: Software for Constructing Population Trees From Allele Frequency Data and Computing Other Population Statistics With Windows Interface.” Molecular Ecology and Evolution 27: 747–752. 10.1093/molbev/msp312 PMC2877541 20022889

[eva70084-bib-0060] Tammi, J. , M. Appelberg , U. Beier , T. Hesthagen , A. Lappalainen , and M. Rask . 2003. “Fish Status Survey in Nordic Lakes: Effects of Acidification, Eutrophication and Stocking Activity on Present Fish Species Composition.” Ambio 32: 98–105. 12733793 10.1579/0044-7447-32.2.98

[eva70084-bib-0061] Taylor, H. R. , N. Dussex , and Y. van Heezik . 2017. “Bridging the Conservation Genetics Gap by Identifying Barriers to Implementation for Conservation Practitioners.” Global Ecology and Conservation 10: 231–242. 10.1016/j.gecco.2017.04.001.

[eva70084-bib-0062] Vlahiotis, K. , C. J. Hogg , A. Moehrenschlager , et al. 2023. Do Conservation Translocations Involve or Result in Hybridization and What are the Consequences of Hybridization for Conservation? A Systematic Review Protocol. OSFPreprints. 10.31219/osf.io/rg59a.

[eva70084-bib-0063] Waples, R. S. , and C. Do . 2010. “Linkage Disequilibrium Estimates of Contemporary Ne Using Highly Variable Genetic Markers: A Largely Untapped Resource for Applied Conservation and Evolution.” Evolutionary Applications 3: 244–262. 10.1111/j.1752-4571.2009.00104. 25567922 PMC3352464

[eva70084-bib-0064] Weir, B. S. , and C. C. Cockerham . 1984. “Estimating F‐Statistics for the Analysis of Population Structure.” Evolution 38: 1358–1370. 28563791 10.1111/j.1558-5646.1984.tb05657.x

[eva70084-bib-0065] Wennerström, L. 2010. Monitoring Genetic Dynamics of Two Released Populations of Brown Trout (Salmo trutta) in a Foreign, Natural Environment. Master Thesis in Population Genetics. Department of Zoology, Stockholm University.

[eva70084-bib-0066] Wennerström, L. , N. Ryman , J.‐L. Tison , A. Hasslow , L. Dalén , and L. Laikre . 2016. “Genetic Landscape With Sharp Discontinuities Shaped by Complex Demographic History in Moose (*Alces alces*).” Journal of Mammology 97: 1–13.

[eva70084-bib-0067] Willoughby, J. R. , A. M. Harder , J. A. Tennessen , K. T. Scribner , and M. R. Christie . 2018. “Rapid Genetic Adaptation to a Novel Environment Despite a Genome‐Wide Reduction in Genetic Diversity.” Molecular Ecology 27: 4041–4051. 10.1111/mec.14726. 29802799

[eva70084-bib-0068] Zhang, Z. , D. P. Bendixsen , T. Janzen , A. W. Nolte , D. Greig , and R. Stelkens . 2020. “Recombining Your Way Out of Trouble: The Genetic Architecture of Hybrid Fitness Under Environmental Stress.” Molecular Biology and Evolution 37: 167–182. 10.1093/molbev/msz211. 31518427 PMC6984367

