# Supplementary material for: Genetic Monitoring of Brown Trout Released Into a Novel Environment: Establishment and Genetic Impact on Natural Populations
Source: Evol Appl. 2025 Feb 27;18(3):e70084. doi: 10.1111/eva.70084 (PMC11868033; doi:10.1111/eva.70084)
Supplement: Supplementary file 1 — Supinfo S1. [file EVA-18-e70084-s001.docx]

**Supporting Information for:**

Genetic monitoring of brown trout released into a novel environment: establishment and genetic impact on natural populations

Anastasia Andersson, Sara Kurland, Sten Karlsson, Nils Ryman, and Linda Laikre

**Table of Contents:**

| **Table S1** | Sampling locality names and geographical coordinates | Page 2 |
| --- | --- | --- |
| **Table S2** | Number of individuals classified as pure (*Q*<0.99) populations A and B among established fish | Page 3 |
| **Table S3** | Pairwise *F*_ST_ among lake and creek localities as well as introduced and native populations | Page 4 |
| **Table S4** | Genetic diversity within lakes and creek localities and introduced and native populations | Page 5 |
| **Table S5a-b** | Main effects ANCOVA of body length | Page 6 |
| **Table S6** | Frequency of immigrants to the two natural creek populations at two points in time | Page 7 |
| **Table S7** | Genetic composition of the two natural creek populations at two points in time | Page 7 |
| **Table S8** | Genetic diversity within the two creek populations at two points in time,  results from significance testing of temporal change, calculations of diversity  retention, and application of indicator ∆*H* (in separate Excel file) | Supplementary Tables.xlsx |
| **Table S9** | Estimates of effective population size of the two creek populations with the  temporal and linkage disequilibrium methods and application of indicator *N*_e_  (in separate Excel file) | Supplementary Tables.xlsx |
| **Table S10** | Estimates of genetic diversity between the two creek populations expressed as  *F*_ST_ for three time periods, results from significance testing of temporal change  in *F*_ST_, and application of indicator ∆*F*_ST_ (in separate Excel file) | Supplementary Tables.xlsx |
| **Table S11** | Sample sizes for extended data used for monitoring frequency of the 50 allele at the *G3PDH-2* locus over time | Page 9 |
|  |  |  |
| **Figure S1** | Linear regression between assignment probability (SNPs) and the distance of the sampled lake to site of release of the introduced populations | Page 10 |
| **Figure S2** | Proportion of spawners within the genetic groups A, B, and AB | Page 10 |
| **Figure S3** | Linear regression between assignment probability (SNPs) and allele frequency of the *50* allele at the allozyme *G3PDH-2* locus | Page 11 |
| **Figure S4** | Comparison of average assignment probability (SNPs) and allele frequencies at the *G3PDH*-2 allozyme locus the seven within lakes | Page 12 |
| **Figure S5** | Frequency of the *50* allele at the allozyme *G3PDH-2* locus over time in three lakes and the two creek localities | Page 13 |
|  |  |  |
| **Appendix S1** | Detailed description of analyses for exploring establishment of the two released populations and their potential introgression into native creek populations | Page 14-17 |
| **Appendix S2** | DAPC analysis | Page 18-20 |

**Table S1.** Names and geographical coordinates of the seven lakes above the waterfall and the two creek localities below the waterfall. Coordinates are given in WGS84.

| **Locality designation** | **Locality name** | **N** | **E** |
| --- | --- | --- | --- |
| Lake 1 | Lake Lilla Bävervattnet | 64.048849° | 14.632384° |
| Lake 2 | Lake Stora Bävervattnet | 64.052875° | 14.666566° |
| Lake 3 | Lake Djuptjärnen | 64.045039° | 14.693775° |
| Lake 4 | Tarn 2 east of Lake Stora Bävervattnet | 64.055856° | 14.687252° |
| Lake 5 | Lake Lillrörtjärnen | 64.072974° | 14.684168° |
| Lake 6 | Lake Hästskotjärnen 655 | 64.038953° | 14.699871° |
| Lake 7 | Lake Haravattnet | 64.027904° | 14.715494° |
| Creek I | Creek Haravattsån Fallet | 64.007374° | 14.728283° |
| Creek II | Creek Haravattsån Nedre | 63.990735° | 14.740550° |

**Table S2.** Number of individuals classified as pure population A and B in the seven lakes above the waterfall using the assignment probability cut-offs *Q*≥0.75 and *Q*≥0.99. The proportion of fish classified as pure was calculated by dividing the total number of fish with *Q*≥0.75 and *Q*≥0.99 by the sample size.

|  |  | **Cut-off *Q* ≥ 0.75** | | |  | **Cut-off *Q* ≥ 0.99** | | |
| --- | --- | --- | --- | --- | --- | --- | --- | --- |
| **Lake** | **Sample size (*n*)** | **Pure**  **population A**  **(*n*)** | **Pure**  **population B**  **(*n*)** | **Proportion pure**  **(*n*_pureA_ + *n*_pureB_)/*n*_sample_** |  | **Pure**  **population A**  **(*n*)** | **Pure**  **population B**  **(*n*)** | **Proportion pure**  **(*n*_pureA_ + *n*_pureB_)/*n*_sample_** |
| Lake 1 | 60 | 0 | 24 | 0.40 |  | 0 | 2 | 0.03 |
| Lake 2 | 59 | 2 | 2 | 0.07 |  | 0 | 0 | 0.00 |
| Lake 3 | 57 | 9 | 0 | 0.16 |  | 0 | 0 | 0.00 |
| Lake 4 | 57 | 5 | 1 | 0.11 |  | 0 | 0 | 0.00 |
| Lake 5 | 60 | 2 | 0 | 0.03 |  | 0 | 0 | 0.00 |
| Lake 6 | 26 | 5 | 1 | 0.23 |  | 1 | 0 | 0.04 |
| Lake 7 | 59 | 10 | 1 | 0.19 |  | 1 | 0 | 0.02 |
| Total | 378 | 33 | 29 | 0.16 |  | 2 | 2 | 0.01 |

**Table S3.** Average pairwise *F*_ST_ (over 96 SNPs; Weir & Cockerham 1984) among populations in the seven lakes and the temporal creek samples. Significance levels were obtained from genepop. All significant *F*_ST_ values are in bold.

|  | Pop A | Pop B | Lake 1 | Lake 2 | Lake 3 | Lake 4 | Lake 5 | Lake 6 | Lake 7 | Creek I  pre-release | Creek II  pre-release | Creek I  1999 | Creek I  2011 | Creek II  1999 | Creek II  2011 |
| --- | --- | --- | --- | --- | --- | --- | --- | --- | --- | --- | --- | --- | --- | --- | --- |
| Pop A | - |  |  |  |  |  |  |  |  |  |  |  |  |  |  |
| Pop B | **0.423** | - |  |  |  |  |  |  |  |  |  |  |  |  |  |
| Lake 1 | **0.245** | **0.071** | - |  |  |  |  |  |  |  |  |  |  |  |  |
| Lake 2 | **0.146** | **0.153** | **0.042** | - |  |  |  |  |  |  |  |  |  |  |  |
| Lake 3 | **0.123** | **0.193** | **0.057** | **0.020** | - |  |  |  |  |  |  |  |  |  |  |
| Lake 4 | **0.128** | **0.214** | **0.074** | **0.020** | **0.016** | - |  |  |  |  |  |  |  |  |  |
| Lake 5 | **0.129** | **0.214** | **0.077** | **0.028** | **0.011** | **0.014** | - |  |  |  |  |  |  |  |  |
| Lake 6 | **0.124** | **0.229** | **0.084** | **0.024** | **0.015** | **0.016** | **0.015** | - |  |  |  |  |  |  |  |
| Lake 7 | **0.103** | **0.210** | **0.064** | **0.023** | 0.004 | **0.017** | **0.012** | **0.018** | - |  |  |  |  |  |  |
| Creek I pre-release | **0.379** | **0.383** | **0.303** | **0.273** | **0.277** | **0.282** | **0.293** | **0.295** | **0.279** | - |  |  |  |  |  |
| Creek II pre-release | **0.257** | **0.274** | **0.178** | **0.159** | **0.166** | **0.168** | **0.175** | **0.161** | **0.167** | **0.212** | - |  |  |  |  |
| Creek I 1999 | **0.314** | **0.331** | **0.229** | **0.196** | **0.202** | **0.209** | **0.216** | **0.216** | **0.200** | **0.042** | **0.159** | - |  |  |  |
| Creek I 2011 | **0.341** | **0.358** | **0.258** | **0.222** | **0.221** | **0.231** | **0.241** | **0.250** | **0.225** | **0.044** | **0.198** | **0.028** | - |  |  |
| Creek II 1999 | **0.261** | **0.280** | **0.173** | **0.153** | **0.163** | **0.167** | **0.171** | **0.158** | **0.163** | **0.227** | **0.005** | **0.164** | **0.207** | - |  |
| Creek II 2011 | **0.216** | **0.247** | **0.133** | **0.105** | **0.110** | **0.112** | **0.118** | **0.104** | **0.111** | **0.210** | **0.013** | **0.145** | **0.184** | **0.008** | - |

**Table S4.** Measures of genetic diversity within baseline populations and contemporary samples. *H*_E_=expected heterozygosity, *H*_O_=observed heterozygosity, *A*_R_=allelic richness, and *A*_n_=average number of alleles per locus; 95% confidence intervals are given in parentheses.

| **Locality** | ***n*** | ***H*_E_ (95% CI)** | ***H*_O_ (95% CI)** | ***A*_n_ (95% CI)** | ***A*_R_ (95% CI)** |
| --- | --- | --- | --- | --- | --- |
| Population A | 110 | 0.229  (0.190-0.268) | 0.248  (0.204-0.292) | 1.656  (1.560-1.753) | 1.639  (1.545-1.734) |
| Population B | 108 | 0.199  (0.159-0.239) | 0.220  (0.175-0.265) | 1.646  (1.548-1.743) | 1.556  (1.462-1.651) |
| Lake 1 | 60 | 0.274  (0.239-0.309) | 0.285  (0.245-0.326) | 1.854  (1.782-1.927) | 1.790  (1.716-1.864) |
| Lake 2 | 59 | 0.281  (0.244-0.318) | 0.293  (0.252-0.334) | 1.844  (1.770-1.918) | 1.783  (1.709-1.858) |
| Lake 3 | 57 | 0.284  (0.246-0.322) | 0.306  (0.263-0.349) | 1.760  (1.644-1.878) | 1.895  (1.831-1.959) |
| Lake 4 | 57 | 0.275  (0.236-0.314) | 0.281  (0.239-0.323) | 1.802  (1.721-1.883) | 1.747  (1.667-1.828) |
| Lake 5 | 60 | 0.271  (0.232-0.310) | 0.283  (0.242-0.325) | 1.833  (1.757-1.909) | 1.756  (1.679-1.833) |
| Lake 6 | 26 | 0.272  (0.233-0.312) | 0.287  (0.243-0.331) | 1.781  (1.697-1.865) | 1.745  (1.663-1.828) |
| Lake 7 | 59 | 0.288  (0.210-0.326) | 0.285  (0.246-0.324) | 1.854  (1.782-1.927) | 1.791  (1.718-1.864) |
| Creek I  pre-release | 135 | 0.208  (0.169-0.248) | 0.209  (0.168-0.250) | 1.719  (1.627-1.810) | 1.606  (1.516-1.696) |
| Creek I  1999 | 60 | 0.242  (0.205-0.279) | 0.234  (0.195-0.274) | 1.844  (1.770-1.918) | 1.741  (1.665-1.816) |
| Creek I  2011 | 60 | 0.218  (0.180-0.256) | 0.208  (0.167-0.249) | 1.854  (1.782-1.926) | 1.677  (1.599-1.755) |
| Creek II  pre-release | 127 | 0.310  (0.274-0.346) | 0.315  (0.277-0.352) | 1.906  (1.847-1.966) | 1.826  (1.760-1.893) |
| Creek II  1999 | 60 | 0.305  (0.268-0.341) | 0.303  (0.266-0.340) | 1.906  (1.847-1.966) | 1.826  (1.761-1.892) |
| Creek II  2011 | 60 | 0.315  (0.279-0.352) | 0.311  (0.273-0.349) | 1.906  (1.847-1.966) | 1.828  (1.762-1.894) |

**Table S5a.** Main effects ANCOVA of body length, **including** the individuals representing the released populations A and B (total *n*=596). Effect sizes are expressed as partial eta-squared; significant *p* values are in bold. Genetic group=classification based on assignment probability to A or B (see text section 2.5 for details).

| **Factors** | **SS** | **df** | **MS** | ***F*** | ***p*** | **Partial eta-squared** |
| --- | --- | --- | --- | --- | --- | --- |
| Intercept | 7999.30 | 1 | 7999.30 | 748.84 | **0.000** | 0.56 |
| Lake | 2274.63 | 6 | 379.11 | 35.49 | **0.000** | 0.27 |
| Sex | 0.76 | 1 | 0.76 | 0.07 | 0.789 | 0.00 |
| Genetic group | 133.94 | 2 | 66.97 | 6.27 | **0.002** | 0.02 |
| Age | 33049.14 | 1 | 33049.14 | 3093.86 | **0.000** | 0.84 |
| Error | 6174.30 | 578 | 10.68 |  |  |  |

**Table S5b.** Main effects ANCOVA of body length, **excluding** the individuals representing the released populations A and B (total *n*=378). Effect sizes are expressed as partial eta-squared; significant *p* values are in bold. Genetic group=classification based on assignment probability to A or B (see text section 2.5 for details).

| **Factors** | **SS** | **df** | **MS** | ***F*** | ***p*** | **Partial eta-squared** |
| --- | --- | --- | --- | --- | --- | --- |
| Intercept | 3319.06 | 1 | 3319.06 | 654.45 | **0.000** | 0.64 |
| Lake | 1143.71 | 6 | 190.62 | 37.59 | **0.000** | 0.38 |
| Sex | 2.94 | 1 | 2.94 | 0.58 | 0.447 | 0.00 |
| Genetic group | 16.59 | 2 | 8.29 | 1.64 | 0.196 | 0.01 |
| Age | 8847.71 | 1 | 8847.71 | 1744.59 | **0.000** | 0.83 |
| Error | 1861.24 | 367 | 5.07 |  |  |  |

**Table S6**. Frequency of immigrants to the two natural creek populations at two points in time. Immigrants from the released populations are defined as fish with assignment probability (*Q*) to populations A or B or both combined ≥0.75. Immigrants from Creek I are fish in Creek II with assignment probability (*Q*) to Creek I ≥0.75. Immigration frequency was estimated by dividing the number of immigrants with the total sample size for each time point.

|  | **Immigrants** | | |  | **Immigration frequency**  **(*n*_immigrants_) / *n*_sample_** | |
| --- | --- | --- | --- | --- | --- | --- |
| **Locality** | **Immigrants from A and B (*n*)** | **Immigrants from**  **Creek I to Creek II (*n*)** | **Natives (*n*)** |  | **From A and B** | **From Creek I to Creek II** |
| Creek I 1999 | 4 | - | 56 |  | 0.07 | - |
| Creek I 2011 | 4 | - | 56 |  | 0.07 | - |
| Creek II 1999 | 0 | 1 | 59 |  | 0.00 | 0.02 |
| Creek II 2011 | 5 | 2 | 53 |  | 0.08 | 0.03 |

**Table S7**. Genetic composition of the two natural creek populations at two points in time after exclusion of fish classified as immigrants (cf. Table S7). Genetic composition is expressed as average assignment probability (*Q*) to introduced populations A and B, and the native populations in Creek I and Creek II prior to release. Introgression was estimated by summing the average assignment probability (*Q*) to populations A and B per sample. Changes in introgression within locality over time was tested using the Wilcoxon matched pairs test.

|  | **Average assignment *Q* (range and excl. immigrants)** | | | |  | **Introgression** | |
| --- | --- | --- | --- | --- | --- | --- | --- |
| **Locality** | **Creek I** | **Creek II** | **Population A** | **Population B** |  | ***Q*_A_ + *Q*_B_** | **Wilcoxon matched pairs test** |
| Creek I 1999 | 0.83  (0.55-1.00) | 0.12  (0.00-0.34) | 0.02  (0.00-0.24) | 0.03  (0.00-0.43) |  | 0.05 | t = 597.00  z = 1.64  p = 0.101 |
| Creek I 2011 | 0.94  (0.58-1.00) | 0.04  (0.00-0.41) | 0.02  (0.00-0.15) | 0.01  (0.00-0.07) |  | 0.03 |  |
| Creek II 1999 | 0.05  (0.03-0.10) | 0.92  (0.70-0.96) | 0.02  (0.00-0.24) | 0.01  (0.00-0.16) |  | 0.03 | t = 444.00  z = 2.40  p = 0.016 |
| Creek II 2011 | 0.06  (0.02-0.73) | 0.86  (0.17-0.95) | 0.04  (0.00-0.60) | 0.04  (0.00-0.43) |  | 0.08 |  |

**Table S8.** ***Separate Excel file (Supplementary Tables.xlsx).*** Genetic diversity within the native creek populations at two points in time, as well as results from significance testing of temporal change, calculations of diversity retention, and application of indicator ∆*H*

**Table S9.** ***Separate Excel file (Supplementary Tables.xlsx).*** Estimates of effective population size of the native creek populations with the temporal and linkage disequilibrium methods and application of indicator *N*_e_.

**Table S10.** ***Separate Excel file (Supplementary Tables.xlsx).*** Estimates of genetic diversity between the native creek populations expressed as *F*_ST_ for three time periods, results from significance testing of temporal change in *F*_ST_, and application of indicator ∆*F*_ST_.

**Table S11.** Sample sizes of the extended allozyme dataset used for monitoring the frequency of the *50* allele at the *G3PDH-2* locus over 36 years in three lakes above the waterfall and the two creek localities below the waterfall (total *n*=11,767). Allele frequencies for each year and locality are presented in Figure S5.

| **Year of collection** | **Lake 1** | **Lake 2** | **Lake 7** | **Creek I** | **Creek II** |
| --- | --- | --- | --- | --- | --- |
| 1980 |  |  |  | 94 |  |
| 1981 |  |  |  | 67 |  |
| 1982 |  |  |  |  | 113 |
| 1983 |  |  |  |  | 103 |
| 1984 |  |  |  | 25 | 100 |
| 1988 | 116 |  |  | 104 | 100 |
| 1989 | 89 | 47 |  | 97 | 99 |
| 1990 | 104 | 64 |  | 95 | 102 |
| 1991 | 103 | 82 |  | 85 | 100 |
| 1992 | 97 | 106 |  | 101 | 89 |
| 1993 | 110 | 96 |  | 84 | 92 |
| 1994 | 106 | 96 |  | 104 | 100 |
| 1995 | 99 | 113 |  | 104 | 104 |
| 1996 | 101 | 110 |  | 92 | 100 |
| 1997 | 106 | 120 | 107 | 96 | 98 |
| 1998 | 107 | 120 |  | 95 | 103 |
| 1999 | 100 | 114 |  | 115 | 106 |
| 2000 | 100 | 93 |  | 101 | 106 |
| 2001 | 103 | 125 |  | 102 | 105 |
| 2002 | 95 | 119 |  | 103 | 99 |
| 2003 | 96 | 114 | 151 | 96 | 101 |
| 2004 | 93 | 92 | 115 | 89 | 102 |
| 2005 | 101 | 130 | 109 | 80 | 103 |
| 2006 | 100 | 109 | 106 | 89 | 145 |
| 2007 | 58 | 106 | 106 | 28 | 102 |
| 2008 | 90 | 83 | 86 | 83 | 100 |
| 2009 | 97 | 114 | 102 | 90 | 81 |
| 2010 | 56 | 71 | 96 | 76 | 99 |
| 2011 | 125 | 109 | 91 | 98 | 120 |
| 2012 | 65 | 92 |  | 72 | 104 |
| 2013 |  |  |  | 80 | 100 |
| 2014 |  |  |  | 81 | 104 |
| 2015 | 89 | 61 |  |  |  |
| **Total** | **2506** | **2486** | **1069** | **2626** | **3080** |

**
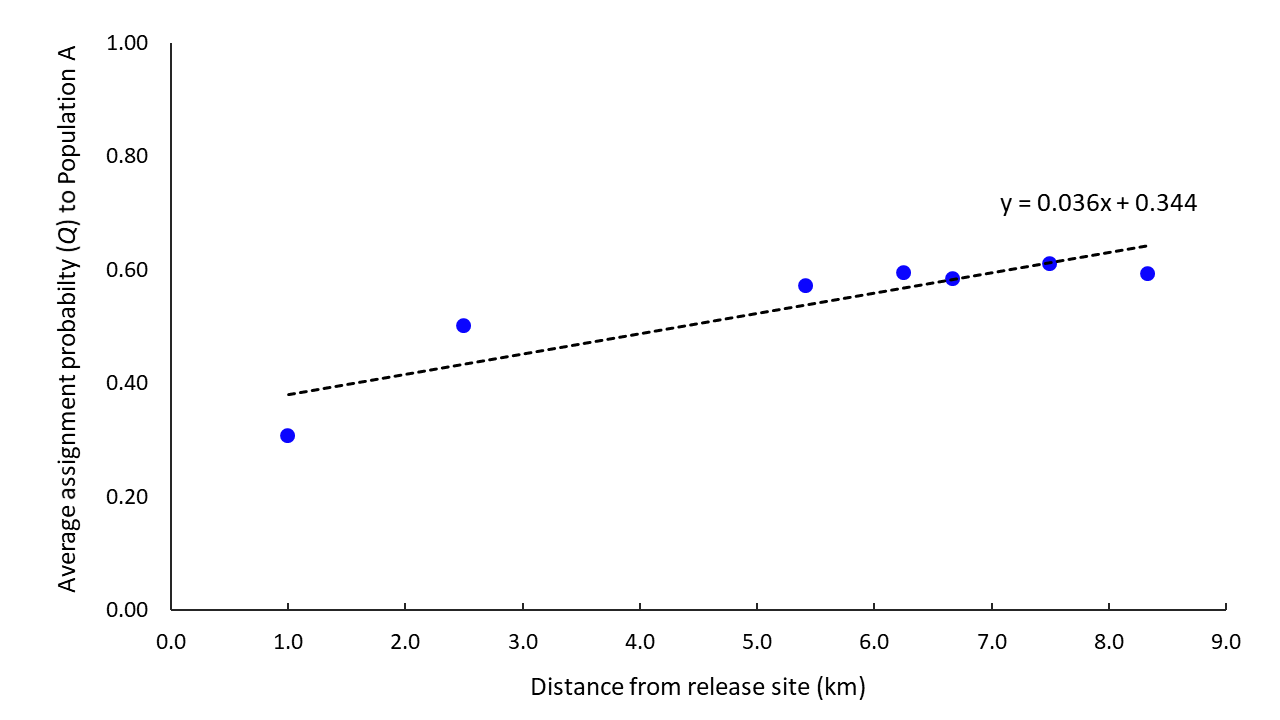
Figure S1.** Relationship between within lake average assignment to population A and the distance of the sampled lake to site of release of the introduced populations (linear regression Pearson *r*=0.89; *p*=0.007).


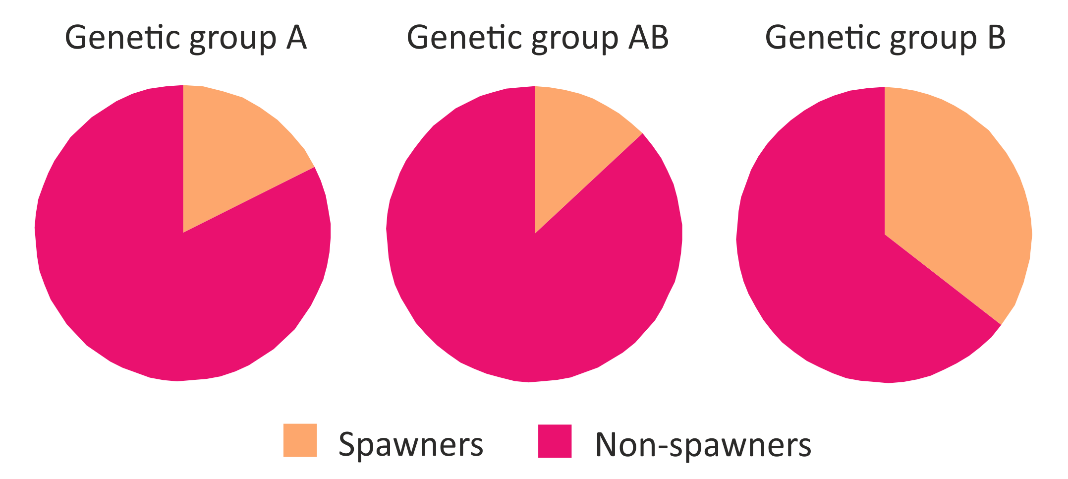


**Figure S2.** Proportion of spawners within the genetic groups A (*Q*_A_≥0.75; *n*=34), AB (*Q*_A_=0.74-0.26; *n*=313), and B (*Q*_A_≤0.25; *n*=31).

**
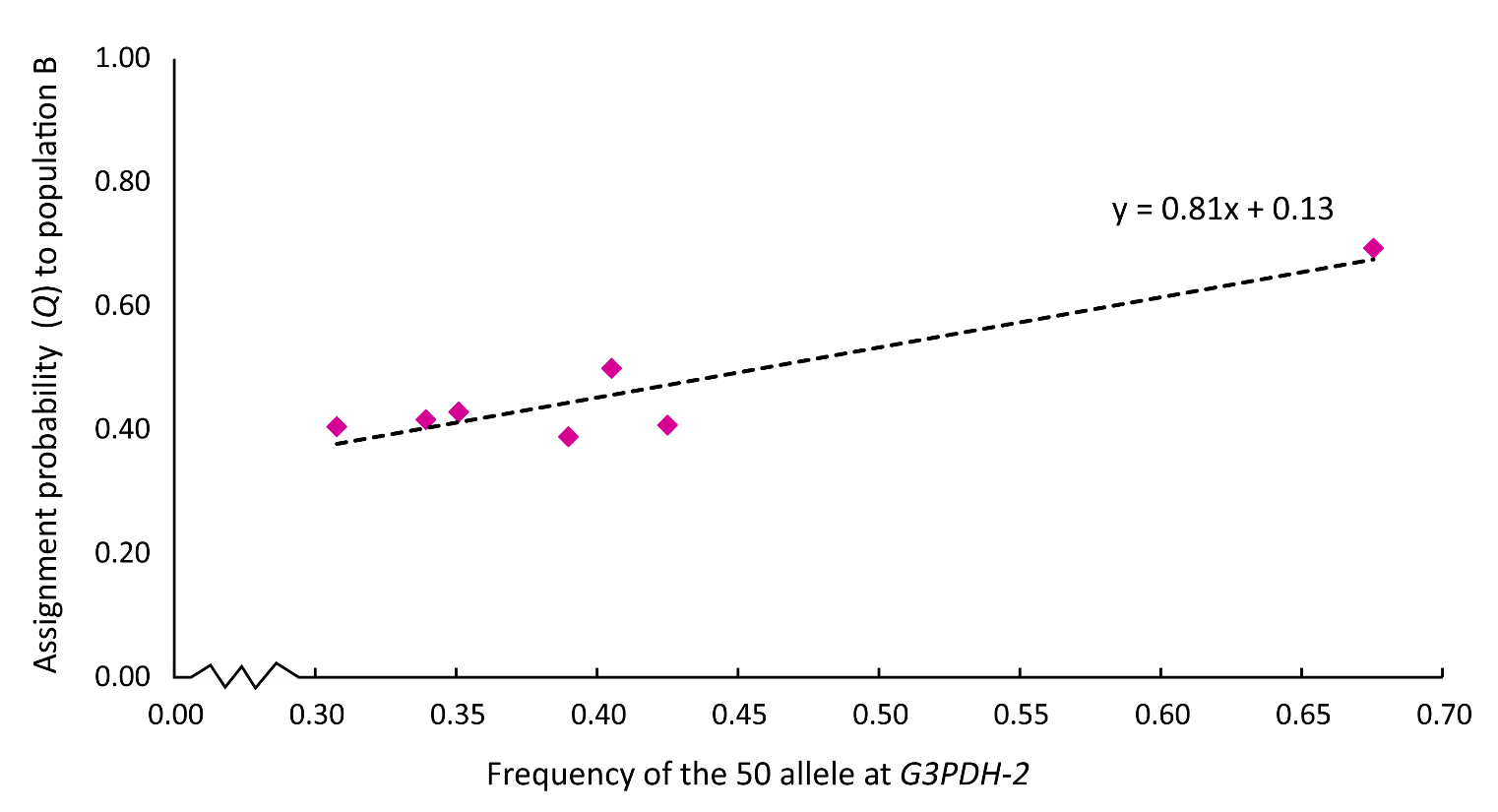
**

**Figure S3.** Relationship between the frequency of the *50* allele at the *G3PDH-2* allozyme locus and within lake average assignment to population B and (*p*=0.003).


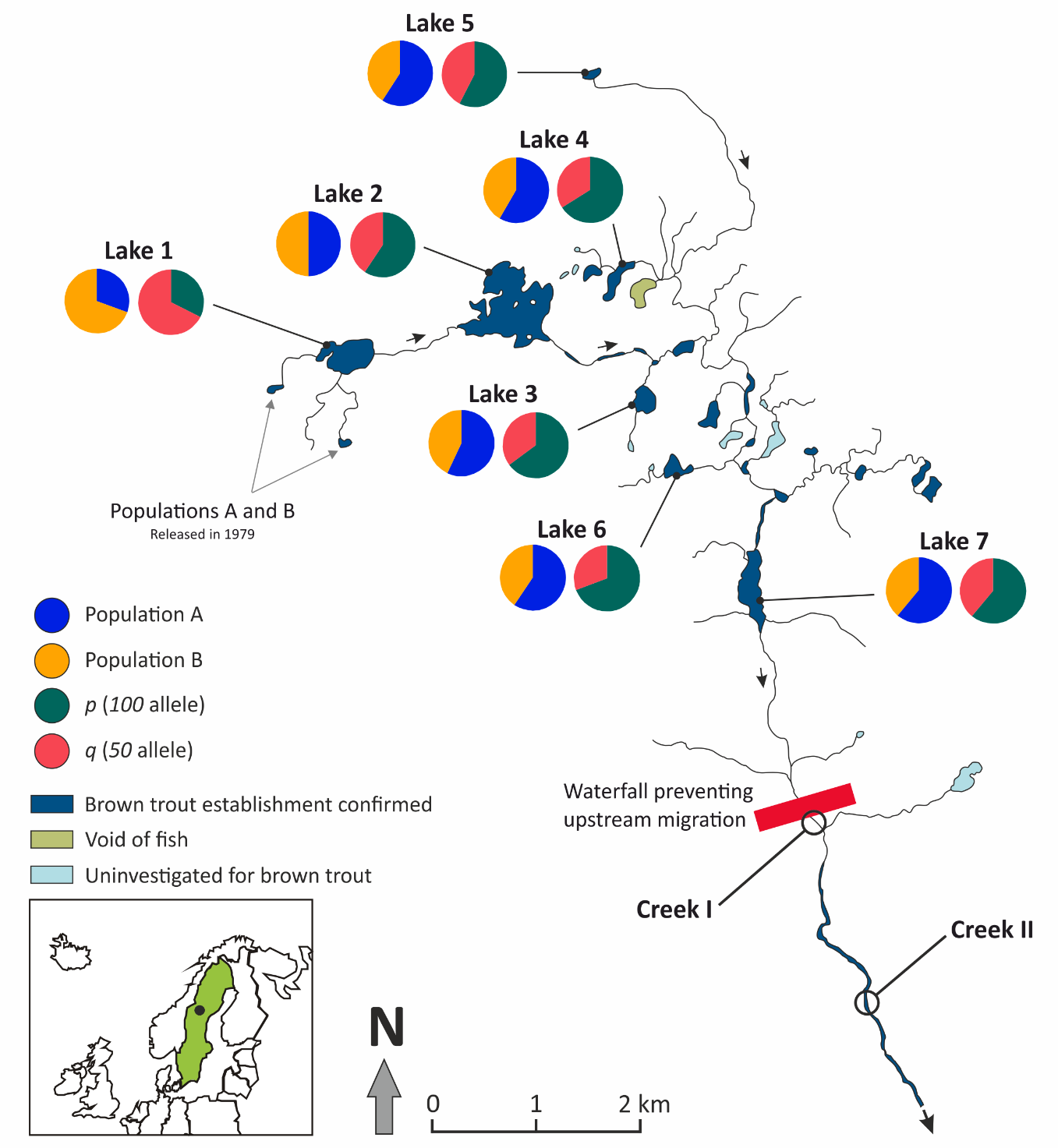


**Figure S4.** Comparison of average assignment probability (*Q*; leftmost pie diagrams) and allele frequencies at the *G3PDH-2* allozyme locus (rightmost pie diagrams) for the same *n*=378 individuals in the seven established populations above the waterfall. Released population A was homozygous for the *100* allele in *G3PDH -2*, while population B was homozygous for the *50* allele. Black arrows indicate direction of water flow.


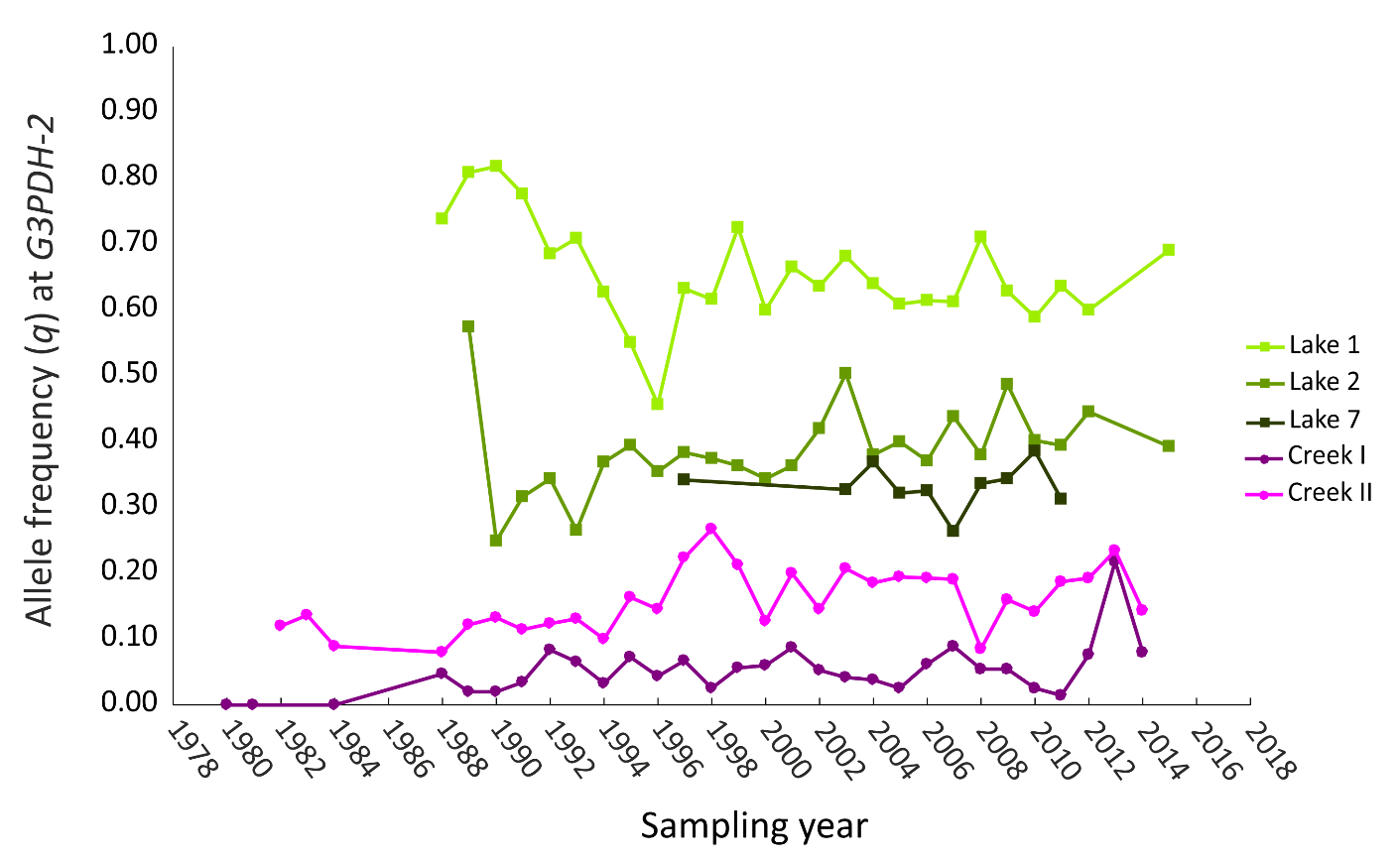


**Figure S5.** Frequency of the *50* allele at the allozyme *G3PDH-2* locus over time in three lakes above the waterfall (cf. Figure 1) and at the two creek localities below the waterfall. Sample sizes are given in Table S11and represent an extended dataset of *n*=11,767 fish collected over a 36 year period and scored for allozymes.

**APPENDIX S1: Defining baselines for released and native populations with SNP data and investigating patterns of establishment and introgression**

**1. Defining baselines for populations A and B using the 96 SNPs**

We checked the classification of the 218 fish selected to represent the baselines for populations A and B (based on their *G3PDH*-2 genotype and birth year) with the 96 SNPs using the structure software (v.2.3.4; Pritchard et al. 2000; Falush et al. 2003). Burn-in length and Markov chains (MCMC) were set as 250,000 steps and 500,000 replicates, respectively. We used the model allowing for admixture and correlated allele frequencies together with an alternative (population-specific) ancestry prior as suggested by Wang 2019 (ALPHA=0.5). We simulated two populations (*K*=2) because previous studies have shown substantial genetic differentiation between populations A and B (*F*_ST_=0.16-0.43) using different genetic markers (Wennerström 2010; Kurland et al. 2022; Table A1). The output from structure was analyzed with structure harvester (v.0.6.94; Earl & vonHoldt 2012). Assignment probability (*Q*), i.e., the mean individual probability of belonging to a certain genetic cluster, was estimated over 20 replicate simulations of the *K* with the clumpp software (v.1.1.2; Jakobsson & Rosenberg 2007).

**Table A1.** Pairwise *F*_ST_ between the introduced populations A and B and the populations Creek I and Creek II pre-release, respectively, using various genetic markers in previous work as well as from the current study. All estimates of *F*_ST_ are statistically significant.

| **Comparison** | **Cohorts** | **Sample size** | **Marker** | **Number**  **of loci** | ***F*_ST_** | **Reference** |
| --- | --- | --- | --- | --- | --- | --- |
| Population A vs B | 1987 and earlier | *n*(A)=211  *n*(B)=494 | Allozymes | 12 | 0.43^1^ | Wennerström 2010 |
| Population A vs B | 1987 and earlier | *n*(A)=50  *n*(B)=50 | Pool-seq | 11,007,131 | 0.16^2^  0.25^3^ | Kurland et al. 2022 |
| Population A vs B | 1987 and earlier | *n*(A)=110  *n*(B)=108 | Allozymes | 15 | 0.43^1^ | Present study |
| Population A vs B | 1987 and earlier | *n*(A)=110  *n*(B)=108 | SNPs | 96 | 0.42^1^ | Present study |
| Creek I vs Creek II | I: 1976-1987  II: 1975-1997 | *n*(I)=636  *n*(II)=1392 | Allozymes | 17 | 0.13^1^ | Palm et al. 2003 |
| Creek I pre-release vs Creek II pre-release | I: 1974-1984  II: 1977-1982 | *n*(I)=135  *n*(II)=127 | SNPs | 96 | 0.21^1^ | Present study |

^1^Weir, B. S., & Cockerham, C. C. 1984. Estimating F-statistics for the analysis of population structure. Evolution, 38, 1358–1370. [https://doi.org/10.1111/j.1558-5646.1984.tb056 57.x](https://doi.org/10.1111/j.1558-5646.1984.tb056%2057.x)

^2^ Nei, M. 1973. Analysis of gene diversity in subdivided populations. Proceedings of the National Academy of Sciences of the United States of America, 70(12), 3321–3323. https://doi.org/10.1073/pnas.70.12.3321

^3^ Karlsson, E. K., Baranowska, I., Wade, C. M., Salmon Hillbertz, N. H. C., et al. 2007. Efficient mapping

of Mendelian traits in dogs through genome-wide association. Nature Genetics, 39(11), 1321–1328.

https://doi.org/10.1038/ng.2007.10

Our results showed that the classification of fish to population A and B with the 96 SNPs corresponds very well with the one based on allozyme data and birth year. All the n=218 individuals were assigned to the correct population (Figure A1) with individual assignment probabilities (*Q*) ranging between 0.99 and 1.00 in both populations A and B.


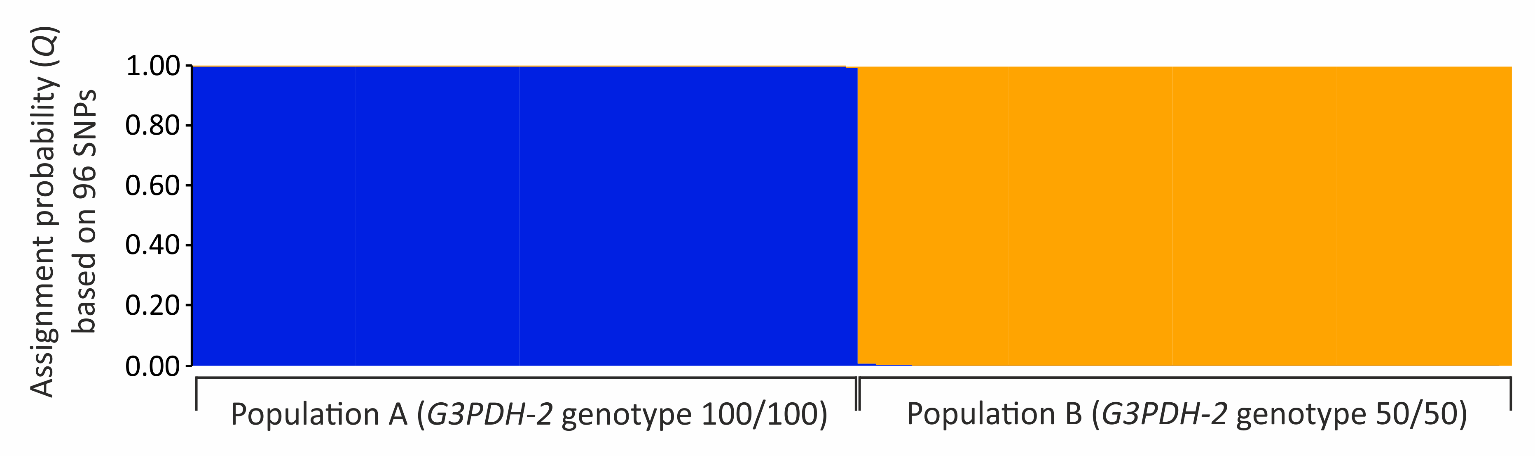
**Figure A1.** structure assignment probability (*Q*) of individual fish from released populations A (*n*=110) and B (*n*=108) based on 96 SNPs. Each individual is represented by one vertical bar on the x-axis.

**2. Establishment of the released populations in the water system**

The establishment patterns of populations A and B were examined by assessing their genetic contribution to the populations currently residing in seven lakes above the waterfall (all located downstream the release site; Figure 1). For this, we used fish collected c. 30 years after the release (during 2007-2015) from these lakes (Table 1); in total, our sample consisted of *n*=378 individuals representing populations descending from the release fish. The *n*=378 descendants were assigned to the baselines for populations A and B (*n*=110 and *n*=108, respectively) in structure, with the same base settings as described in section 1. In this analysis we provided the population origin for the baselines but not for the *n*=378 fish to be assigned, and simulated the number of genetic clusters *K*=1-10. We inferred the most likely number of clusters (*K)* from the parsimony index (*PI*) using kfinder (v.1.0; Wang 2019). The output from structure was analyzed as described in section 1 in this, as well as all subsequent analyses.

**3. Defining baselines for the native populations below the waterfall with 96 SNPs**

We confirmed the classification of fish selected to represent the baselines for Creek I pre‑release (*n*=135) and Creek II pre-release (*n*=127) based on site of collection and birth year with the 96 SNPs in structure. Our hypothesis was that this should be straight forward because Palm et al. (2003) had reported substantial genetic differentiation between the two creek localities using allozymes (*F*_ST_=0.13; Table A1). Thus, we simulated a two-population scenario (*K*=2). We set the burn-in length and Markov chains (MCMC) as 250,000 steps and 500,000 replicates, respectively, and applied the model allowing for admixture and correlated allele frequencies. Similar to the analysis of the released populations, an alternative (population-specific) ancestry prior was used (ALPHA=0.5).

The results from structure showed that all individuals clustered to the correct population with the assignment probability *Q* of at least 0.52 (Figure A2). The assignment probabilities (*Q*) for individuals constituting the Creek I cluster (*n*=135) ranged between 0.52 and 1.00, while *Q* for individuals in the Creek II cluster (*n*=127) ranged between 0.73 and 1.00.


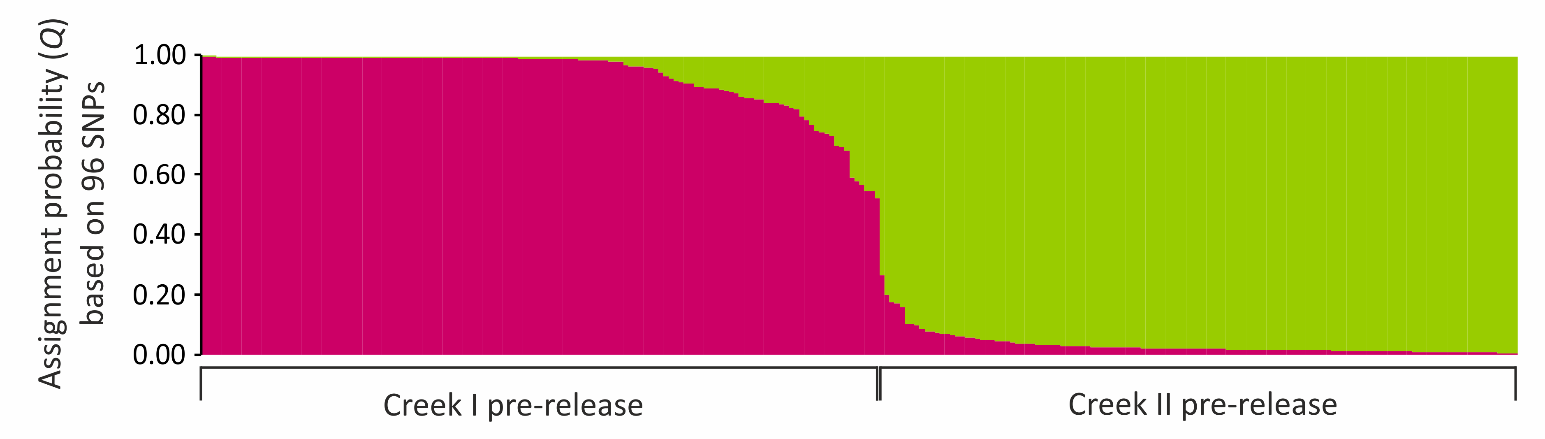
**Figure A2.** structure assignment probability (*Q*) of individual fish from the two native creek populations Creek I (*n*=135) and Creek II (*n*=127) before they were affected by the release based on 96 SNPs. Each individual is represented by one vertical bar on the x-axis.

**4. Investigating genetic introgression of the released populations into the native creek populations**

We examined the potential genetic introgression of released populations A and B into the two creek populations in 1999 and 2011 (total *n*=240) by assigning the fish from the 1999 and 2011 samples to either of the four baselines (Creek I pre-release, Creek II pre-release, population A, or B). The analysis was performed in structure, with the same base parameters as described in section 1. Here, however, we set the alternative ancestry prior ALPHA to 0.25. We simulated *K*=1-10 indicating the population of origin only for the individuals comprising the baselines. The most likely number of clusters (*K)* was inferred from the parsimony index (*PI*), and assignment probability (*Q*) was estimated over 20 replicate simulations of each *K* with the clumpp software.

**References**

Earl, D. A., & vonHoldt, B. M. 2012. STRUCTURE HARVESTER: a website

and program for visualizing STRUCTURE output and implementing the Evanno method. Conservation Genetics Resources, 4, 359–361. [https://doi.org/10.1007/s1268 6-011-9548-7](https://doi.org/10.1007/s1268%206-011-9548-7)

Falush, D., Stephens, M., & Pritchard, J. K. 2003. Inference of population structure using multilocus genotype data: Linked loci and correlated allele frequencies. Genetics, 164, 1567–1587.

Jakobsson, M., & Rosenberg, N. A. 2007. CLUMPP: A cluster matching and permutation program for dealing with label switching and multimodality in analysis of population structure. Bioinformatics, 23, 1801–1806. <https://doi.org/10.1093/bioinformatics/btm233>

Kurland, S., Rafati, N., Ryman, N., & Laikre, L. 2022. Genomic dynamics of brown trout released to a novel environment. Ecology and Evolution, 12, e9050. <https://doi.org/10.1002/ece3.9050>

Palm, S., Laikre, L., Jorde, P. E., & Ryman, N. 2003. Effective population size and temporal genetic change in stream resident brown trout (*Salmo trutta*, L.). Conservation Genetics, 4, 249–264.

Pritchard, J. K., Stephens, M., & Donnelly, P. 2000. Inference of population structure using multilocus genotype data. Genetics, 155, 945–959.

Wang, J. 2019. A parsimony estimator of the number of populations from a STRUCTURE-like analysis. Molecular Ecology Resources, 00, 1–12.

Wennerström, L. 2010. Monitoring genetic dynamics of two released populations of brown trout (*Salmo trutta*) in a foreign, natural environment. Master Thesis in Population Genetics, Department of Zoology, Stockholm University.

**APPENDIX S2: Assessing establishment of introduced populations and their genetic introgression into native populations with DAPC**

We wanted to confirm our results from structure regarding the establishment of the released populations above the waterfall and their genetic introgression into the native population below the waterfall by using an additional approach – a Discriminant Analysis of Principal Components (dapc; Jombart et al, 2010). The dapc is implemented in the adegenet package (v 2.1.5; Jombart, 2008; Jombart & Ahmed, 2011) in R (v.4.1.2; R Core Team, 2021). We assessed the optimal number of genetic clusters (*K*) using the *find.clusters* function, which computes and returns Bayesian information criterion (BIC) score for various values of *K*. The optimal number of clusters may then be selected by visually examining plots of BIC scores. In an ideal scenario, the optimal *K* is represented by the lowest BIC score, though in more complex empirical cases, this may be as straight forward – in these situations the optimal *K* is indicated by an elbow in the plot (Jombart & Collins, 2015).

We used the following script when running *find.clusters*:

> find.clusters(x, clust=NULL, n.pca=NULL, n.clust=NULL, method=c("kmeans"), stat=c("BIC"), choose.n.clust=TRUE, max.n.clust=20, n.iter=1e5, n.start=10, scale=FALSE, pca.select=c("nbEig"), perc.pca=100, dudi=NULL)

Overall, population structure above and below the waterfall detected with the dapc method largely coincided with the findings from the structure analysis. In the material from the lakes above the waterfall, dapc identified three genetic groups as opposed by two detected with structure however (Figure 2a-b). This can be explained by dapc classifying individuals with similar proportions of genes originating from population A and B, respectively, to a separate group as this method does not provide probabilities of belonging to different genetic groups. The average assignment *Q* (obtained from structure) of these fish is around 50% to either of the baseline populations (*Q*_A_=0.56; *Q*_B_=0.44), compared to the individuals classified as Population A (*Q*_A_=0.87) and Population B (*Q*_B_=0.79).

For the dataset including the contemporary samples from the localities Creek I and Creek II, together with the baselines for the released and the native creek populations, dapc suggested four genetic groups (Figure 2d). These groups appear to correspond to the clusters identified in the structure analysis very well (Figure 2c).

**
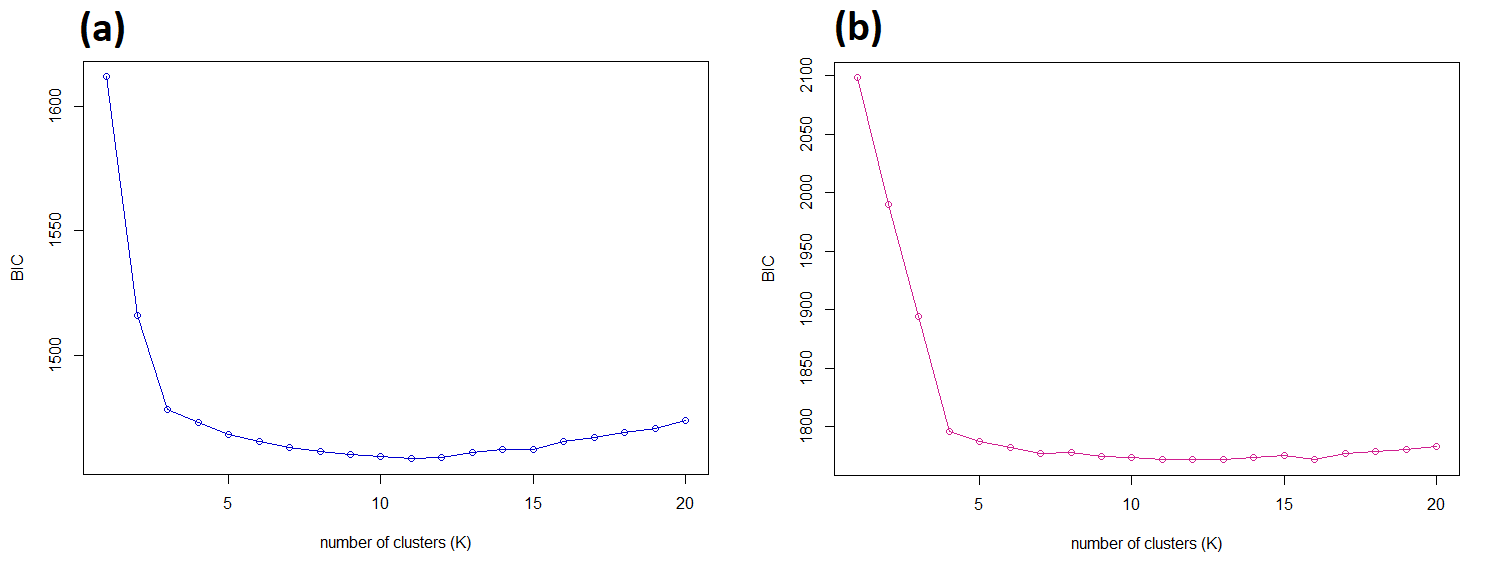
Figure A3.** Plots of BIC score as a function of the number of genetic clusters (*K*) for the datasets including (a) the seven lakes above the waterfall and baselines for the released populations and (b) the two creek localities together with baselines for the released and the native populations.

**
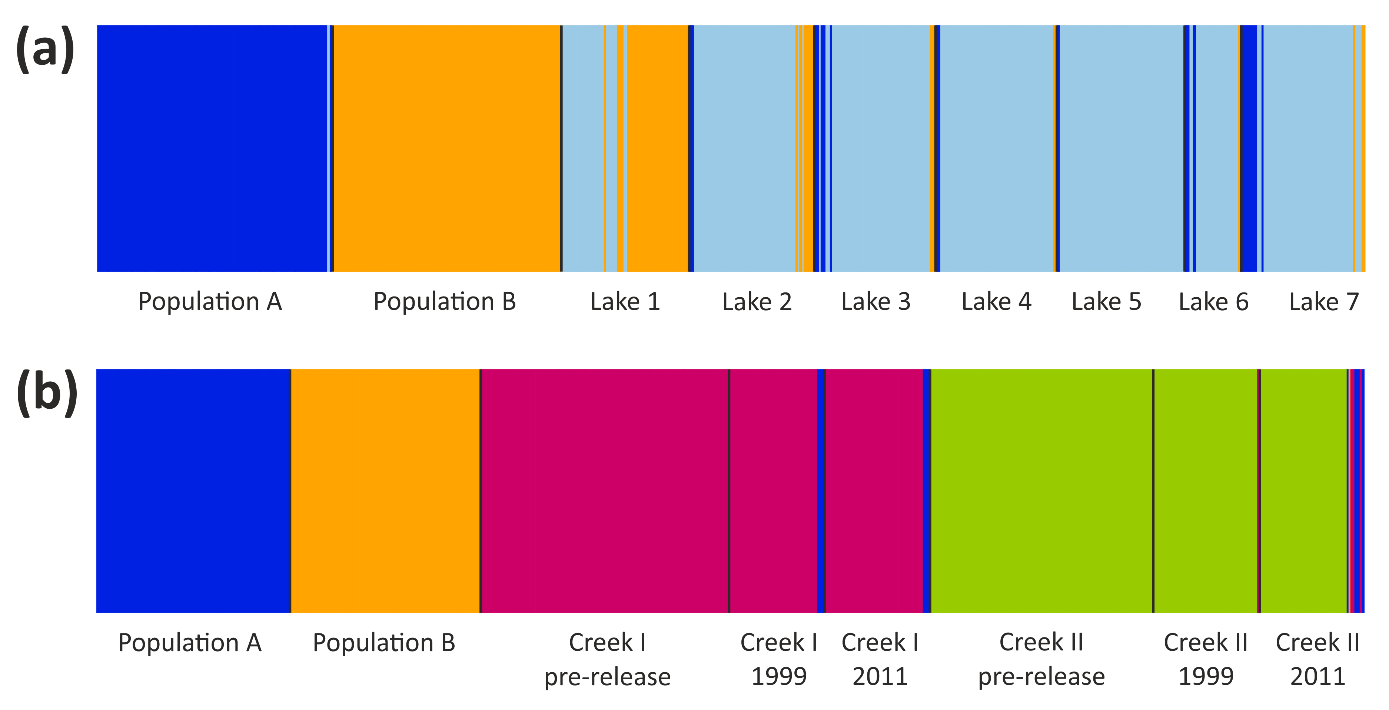
**

**Figure A4.** Plots showing affiliation of individual fish to clusters (populations) using dapc and 96 SNPs, with each fish represented by one vertical bar. The optimal number of clusters (*K*) for the data was determined based on dapc’s Bayesian information criterion (BIC). Panel (a) shows cluster membership of the established released and panel (b) illustrates the natural populations below the waterfall. This figure is included in Figure 2 in the main text.

**References**

Jombart, T. (2008). Adegenet: A R package for the multivariate analysis of genetic markers. Bioinformatics, 24, 1403–1405. doi.org/10.1093/bioinformatics/btn129

Jombart, T., & Ahmed, I. (2011). Adegenet 1.3‐1: New tools for the analysis of genome‐wide SNP data. Bioinformatics, 27, 3070–3071. doi.org/10.1093/bioinformatics/btr521

Jombart, T. & Collins, C. (2015). A tutorial for Discriminant Analysis of Principal Components (DAPC) using adegenet 2.0.0. Imperial College London, MRC Centre for Outbreak Analysis and Modelling. https://adegenet.r-forge.r-project.org/files/tutorial-dapc.pdf

Jombart T, Devillard S, Balloux F (2010). Discriminant analysis of principal components: a new method for the analysis of genetically structured populations. BMC Genetics, 11, 94. doi.org/10.

1186/1471-2156-11-94

Pritchard, J. K., Stephens, M., & Donnelly, P. (2000). Inference of population structure using multilocus genotype data. Genetics, 155, 945–959.

R Core Team (2021). R: A language and environment for statistical computing. R Foundation for Statistical Computing, Vienna, Austria. https//:www.R-project.org/
